# Transcriptional and Microenvironmental Landscape of Macrophage Transition in Cancer: A Boolean Analysis

**DOI:** 10.3389/fimmu.2021.642842

**Published:** 2021-06-10

**Authors:** Ugo Avila-Ponce de León, Aarón Vázquez-Jiménez, Meztli Matadamas-Guzman, Rosana Pelayo, Osbaldo Resendis-Antonio

**Affiliations:** ^1^Programa de Doctorado en Ciencias Biológicas, Universidad Nacional Autónoma de México, Ciudad de México, Mexico; ^2^Human Systems Biology Laboratory, Instituto Nacional de Medicina Genómica (INMEGEN), Ciudad de México, Mexico; ^3^Doctorado en Ciencias Biomédicas, Universidad Nacional Autónoma de México, Ciudad de México, Mexico; ^4^Oncoimmunology Laboratory, Centro de Investigación Biomédica de Oriente, Instituto Mexicano del Seguro Social, Puebla, Mexico; ^5^Coordinación de la Investigación Científica - Red de Apoyo a la Investigación, UNAM, Ciudad de México, Mexico

**Keywords:** macrophage, phenotype, boolean models, systems immunology, gene regulatory network, cancer immunology

## Abstract

The balance between pro- and anti-inflammatory immune system responses is crucial to face and counteract complex diseases such as cancer. Macrophages are an essential population that contributes to this balance in collusion with the local tumor microenvironment. Cancer cells evade the attack of macrophages by liberating cytokines and enhancing the transition to the M2 phenotype with pro-tumoral functions. Despite this pernicious effect on immune systems, the M1 phenotype still exists in the environment and can eliminate tumor cells by liberating cytokines that recruit and activate the cytotoxic actions of TH1 effector cells. Here, we used a Boolean modeling approach to understand how the tumor microenvironment shapes macrophage behavior to enhance pro-tumoral functions. Our network reconstruction integrates experimental data and public information that let us study the polarization from monocytes to M1, M2a, M2b, M2c, and M2d subphenotypes. To analyze the dynamics of our model, we modeled macrophage polarization in different conditions and perturbations. Notably, our study identified new hybrid cell populations, undescribed before. Based on the *in vivo* macrophage behavior, we explained the hybrid macrophages’ role in the tumor microenvironment. The *in silico* model allowed us to postulate transcriptional factors that maintain the balance between macrophages with anti- and pro-tumoral functions. In our pursuit to maintain the balance of macrophage phenotypes to eliminate malignant tumor cells, we emulated a theoretical genetically modified macrophage by modifying the activation of NFκB and a loss of function in HIF1-α and discussed their phenotype implications. Overall, our theoretical approach is as a guide to design new experiments for unraveling the principles of the dual host-protective or -harmful antagonistic roles of transitional macrophages in tumor immunoediting and cancer cell fate decisions.

## Introduction

Macrophages are essential cells in inflammatory responses and immune regulation. However, these cells have heterogeneous functions depending on their polarization. Despite the importance of the process, only the M1 and M2 phenotypes have been experimentally characterized (1, 2). Nevertheless, there are macrophages with intermediate functional phenotypes or hybrid stages that have not been fully described yet. M1, M2, and some hybrid states have specific gene expression and secretion profiles, depending on the microenvironment signals (3). For instance, M1 macrophages express NFκB or STAT1 and secrete TNF-α and IL-12, correlated with a pro-inflammatory response. On the contrary, the M2 macrophages are correlated with an anti-inflammatory response, but their expression profiles are subject to the hybrid stages. As far as we know M2 macrophages have 4 hybrid stages, called M2a, M2b, M2c, and M2d (4). M2a macrophages express STAT6 and secrete IL-10, TGF-β, and IL-1RA, associated with pro-fibrotic functions and inhibition of Th1, and a Th2 response (5). M2b macrophages express Erk and AP-1, and secrete IL-10, TNF- α, and IL-1, correlated with immune regulation (6). M2c macrophages express STAT3 and secrete IL-10 and TGF-β, involved in tissue repair, matrix remodeling, and immunosuppressive behavior (7). Finally, M2d macrophages express HIF1-α or a defective NFκB, enhance STAT1, and induce the secretion of IL-10, TGF-β, and VEGF. M2d is associated with tumors, enhancing the angiogenic process, metastasis, tumor growth, and regulating the immune system (8–10). In principle, hybrid stages in macrophage polarization depend on the microenvironment signals, such as cytokines, membrane receptors, and transcription factors, to determine their specific function and cell fate. However, macrophage hybrid states can transit from one to another depending on the stimuli. M1 macrophages can polarize reversibly into M2a, M2c, and M2d, while M2c can polarize reversibly into M2a (8–11). Moreover, the microenvironment signals can be secreted by other cells within the tissue or by the macrophages themselves (12). Despite the previous characterization, theoretically it has been proposed a continuum transition between the mentioned phenotypes (13). Therefore, integrating the signaling responses and molecular mechanisms involved in the macrophage polarization is required to untangle the complexity of the transitions between the different phenotypes.

As stated above, macrophages respond to tumor microenvironment stimuli in a coordinated and regulated manner. At the molecular level, transcription factors regulate the expression of other transcription factors or mRNA molecules inducing a change in the behavior based on the stimuli. These interactions can be summarized in a gene regulatory network where we can analyze their dynamic properties through mathematical models (14). Given that gene regulatory networks contemplate a large number of variables and the interactions among them, modeling their behavior using a continuous approach would be difficult due to the lack of kinetic parameters. However, gene regulatory networks represent variables as nodes and the interactions between them as edges, making a discrete approach suitable because it requires few parameters. A common approach is the Boolean modeling. This mathematical approach is, where we numerically represent the state of the nodes as 0 (OFF) or 1 (ON). The OFF state means that a transcription factor or molecule is below a certain threshold, meaning it is inactive, while ON indicates an active molecule. The activation and inactivation rules for each node are called Boolean functions. These rules, indicate how each node is activated or inhibited are obtained from experimental evidence and mathematically represented through logical operators: AND (stated as: &), OR (stated as: |), and NOT (stated as: ¬), obtained based on the experimental evidence on how each node is activated or inhibited. The system is solved *via* a synchronous or asynchronous update where we transform a static gene regulatory network into a dynamic one. By applying a synchronous all Boolean functions are updated simultaneously, and they will eventually arrive at a steady state, called an attractor. An attractor is a point where a group of states will converge to that point and they cannot leave this state until a perturbation occurs (15, 16). The biological interpretation of an attractor is a certain cell type or function defined by a gene pattern, it can be related to one or more subtypes of phenotypes and a phenotype can be associated with one or more attractors. This approach has been used to successfully model biological systems (17, 18), and study the cell fate decisions of the adaptive immune response by their specific signals (19, 20). Notably, this approach validated the continuum hypothesis of polarization states by integrating a network of external cytokine signals (21, 22). Although valuable endeavors have recovered and model some of the macrophage phenotypes (M1, M2a, M2b, and M2c) (21, 22). However, there is a lack of information about other phenotypes and their functionalities. We propose a new model capable of recreating the macrophage phenotype diversity considering relevant experimental evidence to fulfill this gap.

In this paper, we present a Boolean dynamical analysis over an updated signaling regulatory network of macrophages to assess the polarization of intermediate phenotypes immersed in the tumor microenvironment. Our Boolean model predicted known macrophage phenotypes, plus hybrid intermediate phenotypes with mixed physiological functions associated with tumor eradication, tissue repair, and progression of tumor cells. Then, we proceeded with an in-depth analysis of the functional properties of the hybrid states. Analyzing the stability of the new hybrid states. As a result, we acknowledge the importance of STAT1, NFκB for the M1 phenotype and HIF1-α for the M2 phenotype. We also proposed a theoretical approach of macrophage immunotherapy in an *in silico* breast cancer microenvironment, which showed promising results in a specific microenvironment towards a better prognosis for cancer eradication. Overall, this work represents a systems biology framework capable of characterizing known macrophage phenotypes and suggesting new hybrid cell populations. Also, it explores the typified macrophages functionality in a tumor microenvironment as well as their robustness and properties. Moreover, our model serves as a computational platform to explore how the tumor microenvironment can potentially modulate macrophages-cancer interaction.

## Material and Methods

### Mathematical Modeling of Network Phenotypes Through Discrete Variables

To explore the feasible space of phenotypes associated with our signaling network, we applied a Boolean approach. Briefly, Boolean approaches assume that each node in the network can be in one of two states (0 or 1), and their dynamic behavior is entirely governed by a Boolean function, which is defined by their regulation. Under this situation, the dynamic state of the i-esime node at time t is given by

$$x_{i}(t+1)\;=\;f_{i}(x_{i1}(t),x_{i2}(t),\;\text{...}\;,\;x_{iki}(t))$$

Where *x_i_* can take the values 0 or 1, and *f_i_* represents the Boolean function of the i-esime node in the network. Note that every node has a Boolean function which determine how that specific node respond to the change of the neighbor regulators (here indicated by the arguments of *f_i_*). Also, *f_i_* maps a Boolean state including multiple Boolean variables at time *t* into a new Boolean state. After one unit of time, the dynamic behavior of the network is obtained when we simultaneously apply the transition function *f_i_* over all the nodes. Starting from the 2^N^ initial states, the Boolean transition functions will allow the network to reach a finite set of states that could cycle between them in a fixed state. These recurrent states are called attractors: if the dynamics reaches a state and stays there, they are called simple attractors, but if they move irregularly in a set of states, they are called complex or loose attractors. All the set of initial states whose transition function guide to the same attractors will form the basin of attraction. The attractors are of great importance because they represent the long-term behavior of the Boolean model and are potentially associated with phenotypes in the system of study (20, 23).

### Analysis and Simulation of the Attractors of Our Boolean Model

A gene regulatory network is a dynamic system. We obtained all of the attractors using the exhaustive search algorithm evaluating the space of 2*^n^* (where n is the number of nodes) initial conditions. So we evaluated 536,870,912 initial conditions using BoolNet Library from R software (24). The obtained attractors carried important biological implications and were associated with macrophage phenotypes. Each attractor was labeled based on experimental evidence. The labeling was done with a function of R package BoolNetPerturb, located at https://github.com/mar-esther23/boolnet-perturb-0.1. To compare the differences between the obtained attractors we reduced the dimension of our variables to plot them in a 2D-space using the t-SNE function in R. Once we had our plot, we clustered them to find similarities between the attractors. To determine the optimal number of clusters, we develop rules based on each possible group value’s associated error, varying from 10 to 30. Finally, the clusters were named based on the functions carried out by the phenotypes of macrophages in each cluster. The model is available in a txt file and an SBML file for it could be easily reproduced. Our Code, data, and implemented analysis can be accessed at: https://github.com/resendislab/M1-M2-Macrophage-Polarization.

### Mutation Analysis

Mutation analysis was divided into two sections: gene deletion and activation. The number of initial conditions for each perturbation is 2*^n^*^–1^ where *n*–1 is the number of nodes minus the perturbed, so for every perturbation we evaluated 268,435456 initial conditions. We permanently set the state of the node as 0 for gene deletion and simulated the dynamics until it reached an attractor. Conversely, gene activation was accomplished by permanently fixing as 1 the state of one node in the network along with all units of time. These considerations were used to mimic the experimental condition of knock-outs or overexpression, respectively. By making this approach, we evaluated the robustness of a given macrophage subtype to resist a gene deletion or activation and how it affects its molecular activation pattern.

In the case of the cell fate map of macrophage polarization, we randomly changed the values of each attractor’s nodes. by a single bit flip. Then we evaluated which nodes changed a given macrophage phenotype into another given phenotype; this perturbation will determine the one-state neighbors.

### Robustness Analysis of Our Model

To validate the robustness of our model, we calculated the Derrida curve and developed the sensitivity analysis of the update rules. A Derrida curve allowed us to evaluate if a gene regulatory network is chaotic, ordered or critical. If the differences between the initial states diverge rapidly from the degrees diagonal, the network is chaotic. To obtain the Derrida curve we proceeded as follows. First, we sampled a random pair of initial states *X1(t)* and *X2(t)* at the same known time *t*. Once we have these states, we calculated the normalized Hamming distance *h(t)* which is the number of bits that differ from the two initial states divided by the number of nodes N. Then, we initialize in time the Boolean network and let both initial states evolve to their next states *X1(t+1)* and *X2(t+1)*, we calculated the normalized hamming distance denoted as *h(t+1)* run the dynamic. We repeated the previous steps for 10 000 random pairs and calculated the average behavior over the realizations. We plotted the value of *h(t)* in the x-axis and the y-axis the value of *h(t+1)*, as well we plotted the diagonal where *h(t)=h(t+1)* which means that the successor states and the initial states are the same. The Derrida curve was calculated with the R package BoolNetPerturb, located at https://github.com/mar-esther23/boolnet-perturb-0.1.

Complementary, the sensitivity analysis showed the differences on the results depending on alterations on each component, either turning on or off respectively. First, we created 50 000 randomly chosen states for each Boolean function define as *f(ki)* where *ki* is the different variables and *i* iterates from 1 to 29. Then, for each of these 50,000 initial states, we created a mutation randomly with only one-bit flip (Hamming distance of 1) labeled as *f(ki’)*. Finally, we applied the update rule for *ki* and *ki’*, and we calculated the sensitivity of the update rule as the fraction of initial states where *f(ki) ≠ f(ki’)*. The sensitivity analysis was carried out using the package of BoolNet in R.

## Results

### Network Reconstruction and Boolean Analysis: The Molecular Basis of Macrophage Polarization in a Tumor Microenvironment

**Figure 1:**
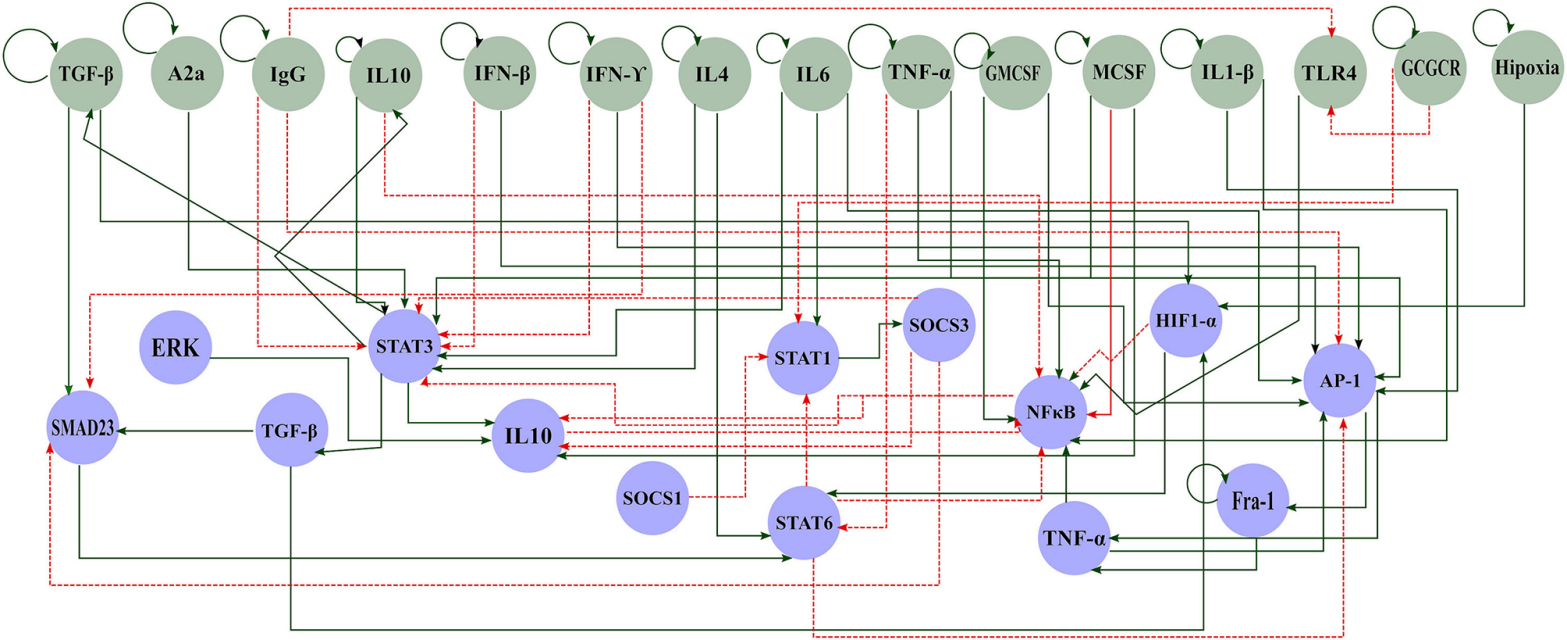
Gene regulatory network of macrophage polarization in a tumor microenvironment. Green circles represent components of the extracellular space and blue circles represent the components of the internal machinery. Solid green arrows represent activation and dashed red arrows inhibition.

The tumor microenvironment contains several cell types, such as cancer cells, immune cells, and mesenchymal cells. These cells produce cytokines that attract other monocytes to the tumor as a natural immune response in the host. The interplay between tumor microenvironment interactions and macrophage functions must be taken into account to understand part of the inflammatory process involved in the interaction between macrophages and cancer. To this end, we reconstructed a signaling network that comprises a variety of regulatory elements associated with macrophage differentiation. We set up our interaction network of the macrophage polarization by a literature search of interactions between external components and how this affects the activation or inhibition of transcriptional factors associated with macrophage polarization. This approach is called a bottom-up methodology. The original reconstruction included 40 nodes. Then, we excluded all those nodes with less than two interactions (in and out) to analyze the dynamic behavior of the network. We did it because these nodes do not contribute information to the dynamic analysis. Therefore, we did the dynamic Boolean analysis onto a network of 29 nodes and 60 interactions. Overall, our transcriptional regulatory network (TRN), integrates extracellular-intracellular components, signal transduction cascades, and transcriptional regulatory mechanisms. This regulatory network of macrophage polarization had two parts, the extracellular components (green nodes) and the master transcriptional factors (blue nodes) that regulate macrophage polarization (**Figure 1** and **Table S1**).

Among the signaling cascades included in our reconstruction, we included the following interactions: Cancer dying cells produce HMGB1 activating Toll-like receptor 4 (TLR4), consequently activating NFκB (25). Briefly, these signals initiate the anti-tumor response in macrophages. However, the macrophage colony-stimulating factor (M-CSF) also triggers NFκB through JAK2 and STAT5. Besides, interferon triggers STAT1, so the cell releases cytokines to inhibit tumor growth (26). On the other side, we also described different activation processes for the M2 sub phenotypes (M2a, M2b, M2c, and M2d). First, M2a activation depends on IL-4 and IL-13 and triggers STAT6 (27). At the same time, STAT6 inhibits STAT1 and NFκB through SOCS1 and KLF4, avoiding the M1 phenotype. Then, M2b activation depends on the signals of AP1 or ERK. AP1 is activated by the presence of IL-1β, while ERK needs the binding to Fc receptors (6). Next, M2c activation relies on the presence of STAT3. IL-10, glucocorticoids, TGF-β, and adenosines trigger STAT3 (28). This cascade induces the secretion of IL-10 and the inactivation of NFκB. Finally, M2d activation hinges on IL-6 or HIF1, related to a hypoxic environment (29). This phenotype is present in solid tumors. This cascade induces the secretion of IL-10, VEGF, and other cytokines. Conversely, the activation of any M2 sub phenotypes culminates in the liberation of cytokines, such as IL-10, that enhance tumor growth. We also included interactions that described M2d phenotype, a tumor-associated-macrophage, and relevant inflammatory cytokines, such as TGF-β and TNF-α. We used this network to dynamically analyze the master regulators that drive the transition between several types of macrophage phenotypes. Hence, to unveil the effect of the tumor microenvironment in macrophage polarization, we performed a Boolean analysis over a reconstructed transcriptional regulatory network for macrophages (**Table S1**). Our network contains a monocyte-derived macrophage regulatory mechanism, as depicted (**Figure 1**). We assumed that all the receptors for the external stimuli were constitutive. This assumption allowed us to eliminate linear interactions and to reduce the size of our network without affecting the dynamic and topology.

### Boolean Modeling of the TRN and Its Spectrum of Macrophage Phenotypes

**Figure 2:**
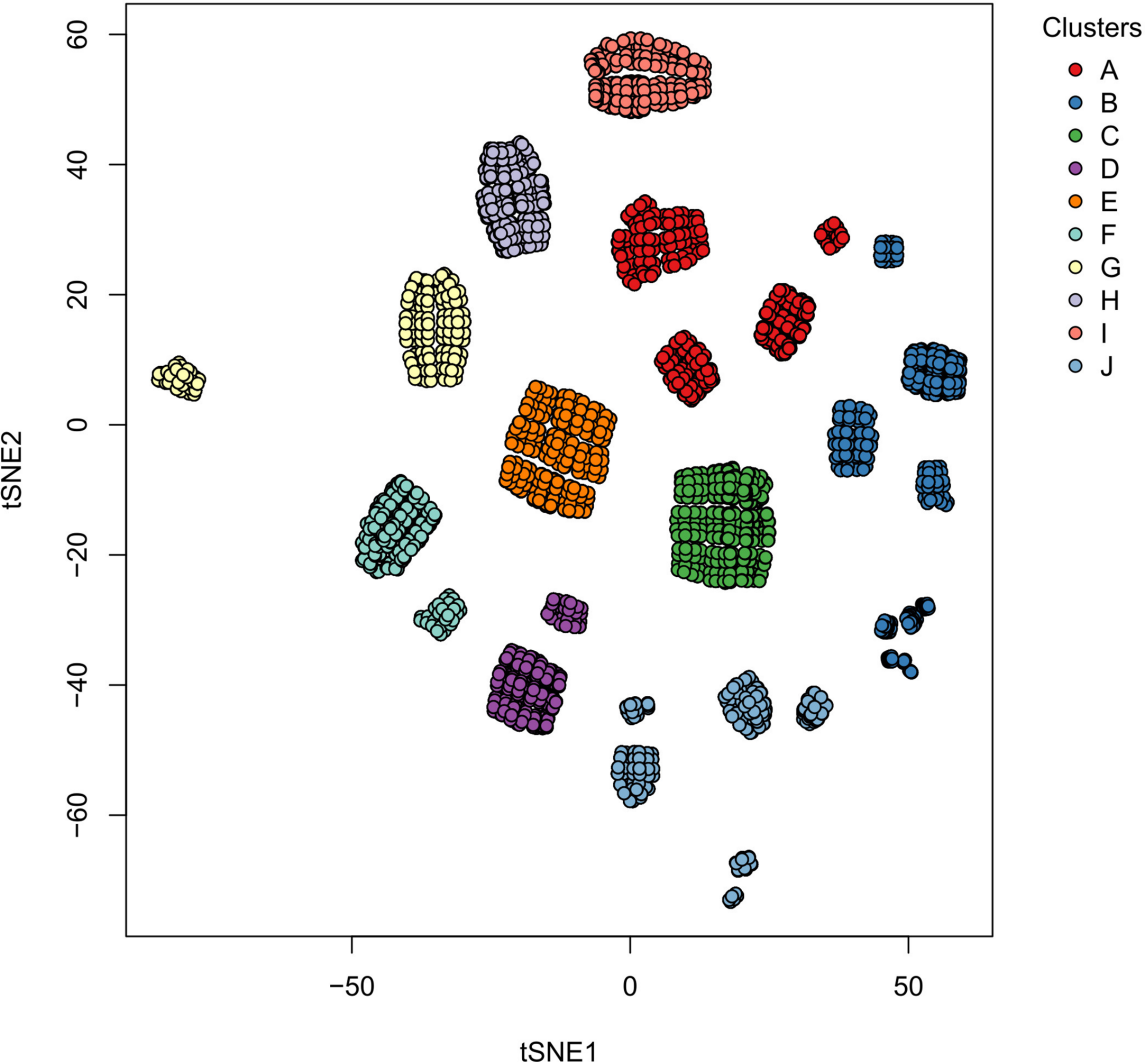
Clusters of the single size attractors obtained in the Boolean simulations. Each circle represents a two-dimensional projection of one attractor, and the color represents a collection of attractors with similar phenotypes between them. The numbers associated with the clusters are explained in **Table 1**.

Based on our reconstructed signaling network, we determined the possible macrophage phenotypes existing in a tumoral microenvironment. Our Boolean model had interconnected signaling pathways, and their dynamics with the external inputs eventually converged to stable states, called single attractors. An attractor is a set of activated or inactivated genes that are time-invariant and potentially represent a macrophage phenotype. We named each attractor based on *in vitro* experimental evidence; we considered six possible steady states associated with a specific macrophage phenotype (**Table 1**). Using a synchronous update, our TRN converged to an entire landscape of 10,430 attractors, where 56 were cyclic, and the remaining were simple attractors. The simple attractors represented 13 phenotypes. Among them, four are already experimentally described (M0, M1, M2b, and M2d). The nine remaining were hybrid phenotypes with two or three macrophage phenotypes. To identify similarities among attractors, we made a bidimensional map using a distributed stochastic neighbor embedding (t-SNE) and a clustering analysis (**Figure S1**). Employing a 30 metrics consensus, we determined that the optimal cluster value was 10 (**Figure S2**). We obtained 10 clusters for 13 phenotypes (**Figure 2**), each one embedding more than one phenotype (**Figure S3**).

**Table 1:** Macrophage phenotypes used to name each attractor.

Phenotype	Transcriptional Factors Activated/External components	Literature Revised
M0	None	
M1	Activation of NFκB, or STAT1, or TNFα and AP1	(26, 30)
M2a	Activation of STAT6	(28, 31)
M2b	Activation of AP1 or ERK	(12)
M2c	Activation of STAT3	(29)
M2d	Activation of TLR4 and A2a or HIF1A	(32)

The attractors obtained after the simulation were labeled based on the macrophage phenotypes reported in the literature.

Among all the phenotypes, seven correlated with a pro-tumoral response and five with an anti-tumoral response. The tumor microenvironment enhances macrophages polarization to pro-tumoral phenotypes with the liberation of interleukins and chemokines. In return, macrophages release growth and immune regulatory factors allowing proliferation and progression of cancer cells. Notwithstanding, in a tumor microenvironment, there are macrophage phenotypes with anti-tumor activity, balancing the elimination of tumor cells without affecting the surrounding tissues.

Due to the massive amounts of stimuli in a tumor microenvironment, we hypothesized that many hybrid activation states exist. We handled macrophage hybrid phenotypes as a single cell identity rather than a heterogeneous population, as in previous works (32). We suggested that a hybrid will be phenotypically distinguished by a combination of expression markers associated with pure M1 and M2 phenotypes. For instance, cluster A contained two-hybrid (mixed macrophage behavior) phenotypes M2bM2d and M2aM2d, macrophages associated with wound healing and regulatory behavior. Based on their composition, we concluded that this cluster was beneficial for tumor growth due to the liberation of growth factors maintaining tumor proliferation and regulatory cytokines to maintain the immune system. On the other hand, clusters B and I consisted of macrophages that would eliminate tumor cells due to the presence of M1, which liberate cytotoxic cytokines to the microenvironment (30). Clusters C and D behavior was complex due to the heterogeneous phenotype composition. Cluster F was a wound-healing and regulatory macrophage due to a hybrid phenotype labeled as M2aM2b (**Table 2**). According to our results, a good scenario against cancer is given by a macrophage state the increases tumor clearance role, by releasing tumor cytotoxic factors, and diminishing the levels of components associated with pro-tumoral behavior.

**Table 2:** Macrophage phenotypes associated with each cluster.

Cluster	Phenotypes	Behavior
A	M2bM2d and M2aM2d	Regulatory/wound healing macrophage
B	M1 and M1M2d	Classical activated macrophages/Pro-tumoral macrophages
C	M1M2bM2d, M1M2bM2cM2d, M1M2b, M2d and M2M2d	Complex behavior. Mixed phenotypes associated with different functions
D	M0, M1M2bM2d, M2b, M2d and M2bM2d	Complex behavior. Mixed phenotypes associated with different functions
E	M1M2bM2d and M2aM2d	Classical activated macrophage/Pro-tumor
F	M2aM2b	Wound-healing macrophages
G	M2aM2cM2d and M2aM2bM2d	Regulatory/wound-healing macrophages
H	M2bM2d and M1M2bM2d	Regulatory/Pro-tumoral macrophages
I	M1 and M1M2d	Classical activated macrophages/Pro-tumoral macrophages
J	M2aM2d and M2aM2bM2d	Regulatory/Pro-tumoral macrophages

The phenotype behavior of each cluster was approximated by the criteria of classification used by (33). Description of the results obtained from the k-means clustering over the t-SNE 2D space.

### Loss and Gain Function Alteration Dictates the Macrophages Subtypes

**Figure 3:**
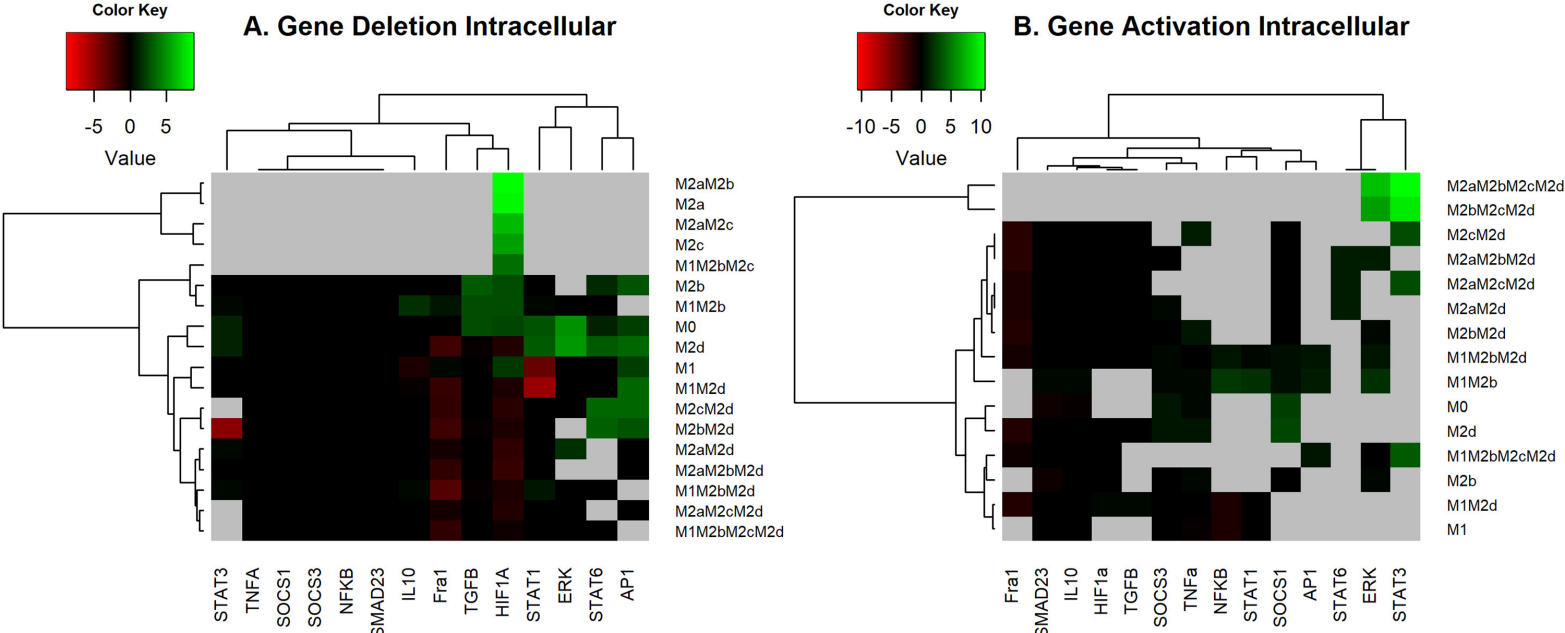
Heat Map of the overexpression and knock-out of transcriptional factors. **(A)** Heat Map of the overexpression of transcriptional factors of macrophage polarization. We maintained the expression of the node as 1, simulated, and reviewed the attractors obtained. All overexpression was compared with the wild-type (original network) with log2 fold change. **(B)** Heat Map of Knock-outs of transcriptional factors of macrophage polarization. The nodes were permanently fixed with a value of 0; the perturbations were realized one by one until the attractors were reached. We compared the phenotypes of the wild-type with the perturbations by a log-fold change. Green and red regions indicate those attractors whose size of the basin of attraction increased or decreased after the perturbation. In black, we denote those attractors with few effects in the basin attraction size versus WT and after perturbation. Gray areas indicate those attractors that exist in the wild-type but do not remain perturbed.

In this section, we computationally assessed the importance of each node in the stability of the phenotypes obtained. For this purpose, we performed a permanent deletion (set the value of the node to zero) or activation (set the value of the node to one) of each node in the dynamic analysis of the TRN. To quantify the effect produced by these perturbations, we applied the following equation:

$$log_{2}\left(\frac{M\pm i}{WT_{i}}\right)$$

where *WT_i_* denotes the size of the basin of attraction for the “i” attractor without alterations, and *M* ± *i* denotes the basin of attraction for the “i” attractor for the deletion(-) and activating (+) of the node. As expected, the previous equation is constrained to those cases where *WT_i_* ≠ 0 and both variables are positively defined. The size of the basin of attraction for WT is depicted in **Figure S4**. **Figure 3** shows the log-fold of the size of the basin of attraction when we compared perturbed and unperturbed (WT) genes stated by columns. Gray areas indicate attractors that were in the wild-type but not in the perturbed state. Attractors with an M1 macrophage will be an anti-tumoral phenotype, and with the M2 macrophage, a pro-tumoral phenotype (rows). Each column is an activation or inactivation of a transcription factor.

In terms of the results, we observed that permanent activation of AP-1, STAT1, or NFκB was very similar because they induce hybrids with tumoricidal capacity (M1). AP-1 and STAT1 activation decreased at 100% pro-tumoral phenotypes and 20% anti-tumoral phenotypes. These transcriptional factors may recover the balance towards a tumor eliminating scenario in the tumor microenvironment (gray areas **Figures 3A** and **S5, S6** respectively). NFκB permanent activation maintained the same behavior as previous transcription factors because it diminished at a 100% pro-tumoral phenotype and 20% anti-tumoral phenotypes, creating a perfect balance of tumor elimination and recovery of the tissue due to the secretion of cytotoxic interleukins and interferons (gray areas in **Figures 3A** and **S5, S6** respectively). On the other hand, constitutive expressions of HIF1-α or TGF-β eliminated pure M1 macrophages (**Figure 3A**) and decreased the available profiles that converged to hybrids with M1 as a component. HIF1-α and TGF-β promoted the development of malignant behavior in macrophages. Once activated, HIF1-α decreased 22% anti-tumoral phenotypes (**Figure S6**) and 40% pro-tumoral phenotypes (**Figure S5**). We concluded cells should avoid the presence of HIF1-α in a tumor microenvironment. Also, TGF-β had the same behavior as HIF1-α (**Figures S5, S6**, respectively), so cells should avert its secretion to revert cancer proliferation. Nevertheless, we hypothesize that the modulation of the balance to anti-tumoral phenotypes could be sufficient to diminish TGF-β.

Conversely, constitutive expressions of NFκB or STAT1 kept M1M2b and M1M2d phenotypes, respectively. Both hybrid phenotypes with well-known tumoricidal capacity. Interestingly, the overexpression of NFκB and STAT1 could be a potential therapeutic strategy because they promote phenotypes that could balance the behavior of macrophages in a tumor microenvironment, favoring tumoricidal capacity (**Figure 3A**).

On the other hand, we found an imbalance in the size of the basin attractors when we knocked out some transcriptional factor expressions separately (**Figure 3B**). Our *in silico* analysis allowed us to conclude that if we knocked out the expression of Fra-1, the M1 and the hybrid M1M2b produced a slight augment in their size of the basin of attraction, implementing a tumoral eradication capacity. Also, we observed that the lack of Fra-1 did not influence the development of the M2d phenotype. Turning off HIF1-α increased phenotypes associated with tumoricidal capacity (M1 and M1M2b), denoting the importance of not expressing these transcriptional factors for eliminating tumor cells (**Figure S7**). However, only affecting HIF1-α activity is insufficient because it enhances pro-tumoral phenotypes at 36% for the wild type macrophage (**Figures 3B** and **S8**). Thus, we had a typical host defense by activating the gamma receptors and liberating IFN-γ to the microenvironment. Notably, unlike gene activation analysis, we observed the emergence of some pure phenotypes, M2a, and M2c, tending to favor tumor progression when we knocked out HIF1-α.

Furthermore, we noted that knock-out of STAT3 decrease the proportion of phenotypes specific for tumor eradication and hybrid with anti-tumoral behavior at 33% (**Figure S7**). Inactivating STAT3 diminished 63% of pro-tumoral phenotypes involved in the regulation of the immune system (M2c) by activating NFκB (**Figure S8**). STAT6 knock-out does not affect anti-tumoral phenotypes (**Figure S7**), but it decreases the attraction basin of phenotypes. M2c phenotype releases IL-10 into the microenvironment creating a scenario for tumor evasion and enhancing tumor metastasis. In agreement with these findings, biological evidence suggests that the inactivation of STAT3 and STAT6 transcriptional factors are associated with reducing tumor growth and metastasis in a model of breast and lung cancer (31, 34).

### Macrophage Polarization Develops Feedback With Microenvironments

Tumor microenvironments shape the polarization of macrophages. To evaluate how the microenvironmental signals alter this process, we analyzed six different signaling environments associated with macrophage phenotypes: M0, M1, M2a, M2b, M2c, and M2d states. We simulated signaling microenvironments activating permanently known profiles of cytokines and signaling metabolites associated with each macrophage phenotype (see **Table 3**) (33, 35).

**Table 3:** Microenvironments associated with specific macrophage subtypes.

Macrophage Phenotype	Signals
M0	No external stimuli
M1	IFN-γ and IFN-β
M2a	IL4 and TGF-β
M2b	IgG and glucocorticoids
M2c	IL10 and IL6 and MCSF
M2d	Adenosines and hypoxia and glucocorticoids

On the right side of the table, we have the phenotypes and the nodes that were kept on during the simulation until an attractor was reached. Simulation of microenvironmental cues for the six macrophage phenotypes modeled in our gene regulatory network.

**Figure 4:**
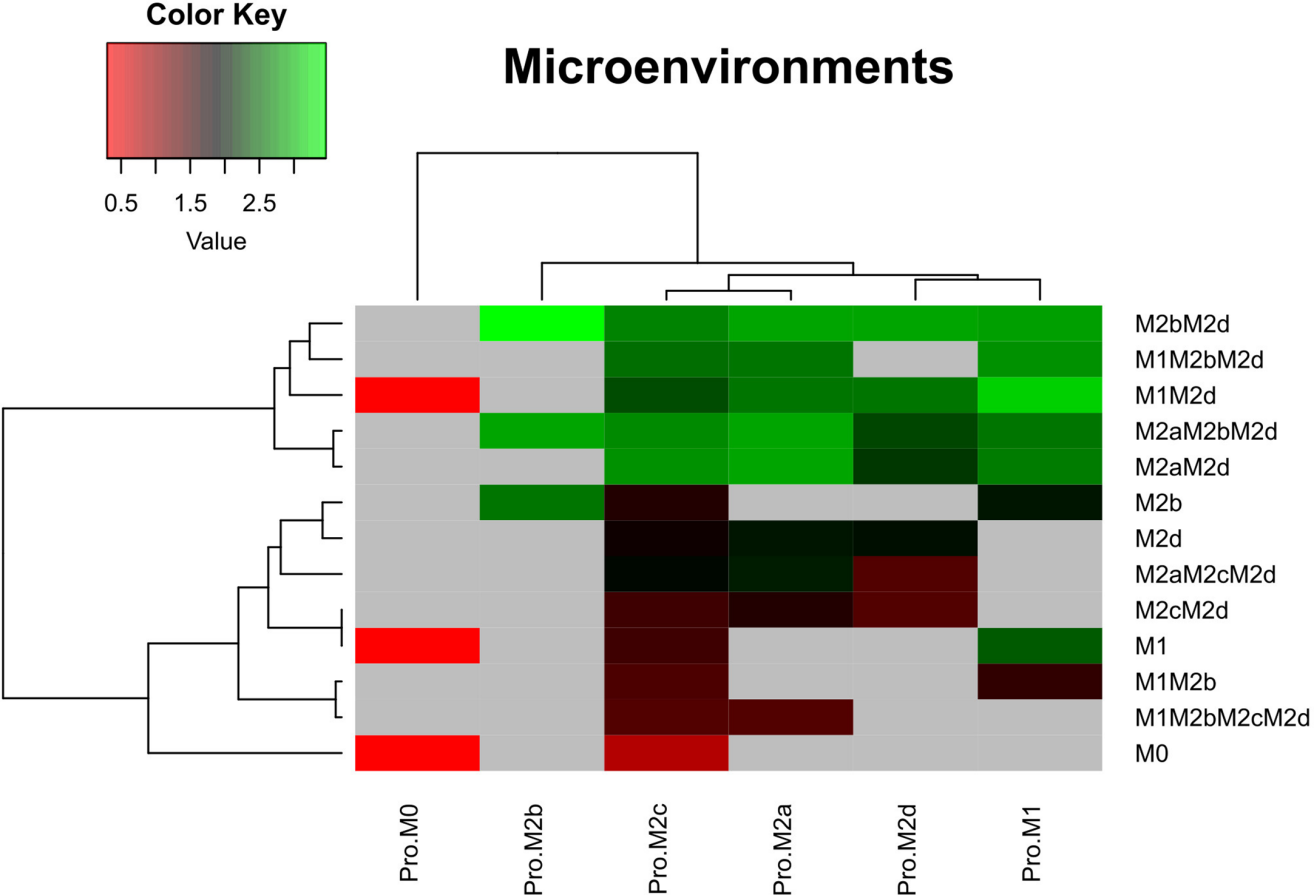
Heat maps of the microenvironments of macrophage polarization. Microenvironments associated with the six phenotypes evaluated in this work. For these simulations we used the criteria of **Table 2**. Once the attractors were obtained, we applied a logarithmic transformation on the size of the basin of attraction for each phenotype. Red stands for a low basin of attraction and green for a high basin of attraction. Pro means we modelled the polarization in a microenvironment adjuvant for each macrophage subtype. M0, monocytes.

**Figure 4** depicts the logarithm of the basin of attraction of the attractors obtained in said microenvironments. Gray areas indicate that the phenotype was not present in the specific microenvironment. Notwithstanding that the Boolean model is a simplified analysis, we highlight a global behavior over how the macrophage phenotype is shaped. For instance, the monocyte microenvironment (Pro-M0) tends to induce three phenotypes at a low rate: M0, M1, and M2d. Fra1 may give a possible explanation underlying the increment in the size of the basin of attraction for M2d. The upregulation of Fra1 ultimately activates NFκB, which in turn will induce the M1 macrophage phenotype.

Furthermore, our computational analysis suggests that the Pro-M1 microenvironment diminishes regulatory and wound-healing phenotypes and enhances tumoricidal capacity (**Figure 4**). In counterpart, Pro-M2b microenvironment decreases tumoricidal capacity, favoring macrophages phenotype to tumor progression and immune regulation. This microenvironment only induces phenotypes either hybrid or simple associated with M2b. On the other hand, M2c microenvironments maintain hybrid phenotypes associated with tumor proliferation and wound-healing components, like angiogenesis. Besides, we noted that the regulatory cytokines like IL-10 and IL-6 in the microenvironment Pro-M2c diminished all possible cytotoxic hybrid activity in macrophages. This finding can explain cytotoxic activity in a pro-tumor microenvironment (36). However, the M2 population was preferred over M1 because they had specific microenvironment components (Pro-M2c) to maintain this imbalance (**Figure 4**). Furthermore, the M2d microenvironment does not generate any macrophage associated with tumoricidal capacity; it only activates macrophages implicated in wound-healing (angiogenesis and tissue recovery) and phenotypes regulating the actions of the immune system in eliminating tumors.

Lastly, macrophage phenotypes were susceptible to local microenvironments inside a tumor. These microenvironments can induce heterogeneity over the composition enhancing specific subtypes of macrophages. We believe that these phenotypes will increase their stability due to the continuum expression of their external signal. However, anti-tumoral behavior occurs even in the activation of pro-tumoral behavior by external signals but with lower stability. This type of modeling scheme may help develop therapeutic strategies to ensure stability despite the microenvironment acting against the treatment of a specific cancer type (37, 38).

### The Importance of Molecular Components in Determining Cell Fate Macrophages

**Figure 5:**
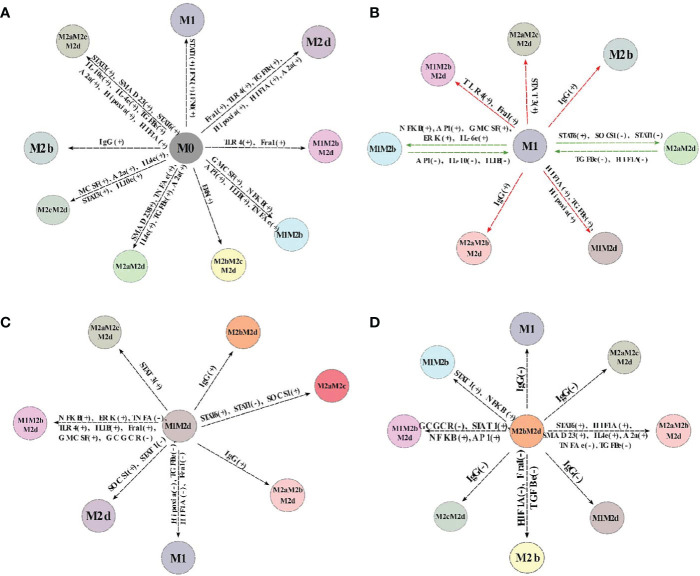
Cell fate map of the macrophage polarization. Of all the attractors obtained, we changed the node’s value and maintained this perturbation until an attractor was reached. If the attractor’s transition to another phenotype, we represent it with a line and the new phenotype obtained by the perturbation. Plus sign (+) means the node was turned off, and we turn it on, while minus sign (-) means the perturbation was on, and we turned off. **(A)** Cell fate map of monocyte (M0). **(B)** Cell fate map of M1 macrophage. **(C)** Cell fate map of M1M2d macrophage. **(D)** Cell fate map of M2bM2d macrophage. Colors represent different states of macrophage polarization.

We analyzed each attractor’s stability previously obtained through a gene perturbation analysis to evaluate the plasticity, closeness, and possible transition between the macrophage subtypes. As shown previously, these sets of analyses are beneficial to describe and uncover the network’s global properties. We obtained a global landscape of the possible transitions among them (**Figure 5**). To accomplish this, we altered each node’s state in the network. If the value of the node was 1, we changed to 0 and vice versa. Then we evaluated if this modification affected the stability of the phenotype in the network. Notably, we observed that only some transitions were allowed among attractors; besides, these transitions depended on different genetic alterations. As expected, phenotypes with the largest basin of attraction were more stable to perturbations (**Figure S4**). This study allowed us to conclude that M0 (monocyte) had a low stability, and it was prone to become other macrophage phenotypes under perturbations (**Figure 5A**). For example, M1 can differentiate from M0 through the activation of STAT1, IFN-γ, or IFN-β, but M1 can re-polarize to M0 (monocyte) activating the inhibitory function of SOCS1. These polarizations were irreversible, which means once they shift to the new phenotype, they cannot shift back to monocytes (**Figure S9**).

On the other hand, M2b transitioned from M0 by activating immunoglobulin G (IgG). B cells liberate IgG and can be cancer-derived (39, 40), so the M2b phenotype can be induced directly from monocytes and not necessarily has to pass first from a TLR4 activated macrophage and then shift to M2b (7). The transition from M0 to M2d was obtained by activating HIF1α or TGF-β or adenosines leading to the inactivation of any transcriptional factor of cytotoxic behavior; this transition was reversible (**Figure S9**). Notably, our simulations allowed us to postulate that the more complex phenotypes, the hybrids, can be obtained from M0. For instance, the tumoricidal/regulatory macrophage given by M1M2b obtained by activating AP1, NFκB, and IL1-β, had an, irreversible transition.

Another interesting observation is that the cytotoxic macrophage named M1 was stable for most perturbations (**Figure 5B**). For example, when we turn on AP1, the M1 phenotype becomes a hybrid phenotype (M1M2b) that maintains its cytotoxic capacity combined with a regulatory behavior. This shift is reversible macrophages can return to an M1 phenotype by inactivating AP1 (**Figure S9**). Furthermore, M2b attained from M1 by activating IgG, stating the importance of the immune complexes for selecting this trajectory. It does not need to be first a TLR4 activated macrophage to promote the transition to the M2b phenotype (7). Hybrid phenotype M1M2d (**Figure 5C**) can favor tumor clearance and recuperation of tissue. Based on our model, we observed this phenotype constrain the macrophage into a hypoxic condition, permanently activating TGF-β. The M1 phenotype can be obtained from M1M2d, turning off hypoxia or TGF-β, but both extracellular conditions are present most of the time in a tumor microenvironment. In this context, pursuing a hybrid phenotype with theoretical benefits such as M1M2d seems to be a better strategy than the induction of the M1 phenotype in a tumor microenvironment. We also observed that M1M2d, a tumoricidal/regulatory state, can shift to the M2d macrophage stage. This polarization is reached by turning on SOCS1 (inhibiting STAT1) or turning off STAT1 (M1 transcriptional factor). Notably, perturbation analysis suggests the emergence of a new phenotype from M1M2d; a hybrid labeled M2aM2c with an irreversible phenotype (**Figure S9**). M2aM2c has a regulatory/pro-tumoral behavior favoring tumor growth and tumor evasion, and it is promoted by turning on STAT6 from M1M2d. M2bM2d has more stability compared with other phenotypes (**Figure 5D**). IgG is a crucial factor that dictates this phenotype’s behavior, so turning off this node, the hybrid phenotype can shift irreversibly to various tumoricidal macrophage stages, like M1 (**Figure S9**). M2b phenotype derived from M2bM2d by turning off HIF1α and the expression of ERK, which permits the secretion of IL-6, a pleiotropic cytokine and immune regulator of IL-10. Finally, turning on STAT1 or NFκB was sufficient to transit to a tumoricidal/regulatory phenotype from M2bM2d, labeled as M1M2b.

Lastly, there is growing evidence that plasticity is a property in the immune system’s response altogether, here we supplied evidence that the response of macrophages is not the exception. Even though macrophages polarize depending on external factors, they can be manipulated to influence the outcome of their external signals. This analysis allowed us to identify potential genetic control points like NFκB and HIF1α, which could serve as potential molecular targets against cancer by modifying the macrophage phenotypes.

### HIF1-α and NFκB as Potential Transcriptional Factors for a Theoretical Treatment Based Approach

Despite tumor microenvironment complexity and variability, their modulation is an appealing strategy to reduce the cancer cell phenotype (41). For instance, in our model, the most relevant activated transcription factors for the polarization to M1 phenotype and M1 hybrid are STAT1 and NFκB; these findings agree with a previous report (42). On the contrary, activation of HIF1-α is associated with the reduction of anti-tumoral macrophages by enhancing pro-tumoral macrophages. We concluded that the inactivation of HIF1-α and activation of NFκB or STAT1 might be critical to shift the balance to an anti-tumoral microenvironment rather than a pro-tumoral.

**Figure 6:**
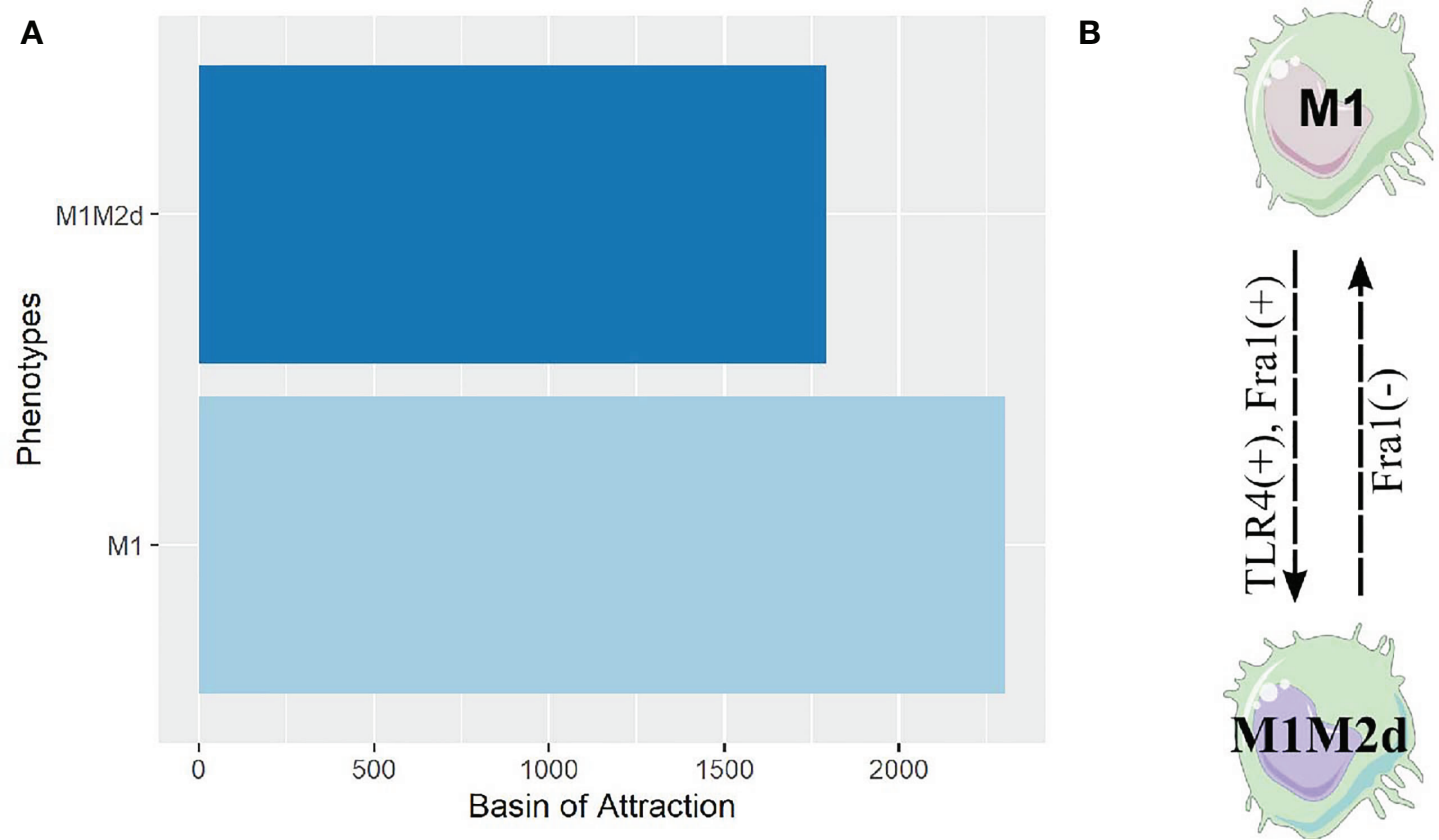
Attractors and cell fate map of our TGEM. **(A)** Bar plot of the attractors obtained from our theoretical genetically modified macrophage. For this analysis we set the value of NFκB to 1 and HIF1-α to 0, and simulated until we obtained these attractors. **(B)** Cell fate map of our theoretical genetically modified macrophage. By analyzing the plasticity of phenotypes through single gene perturbation of the genetically modified macrophage, we obtained the rules of genetic perturbation that contribute transition between macrophages phenotypes. Here (-) means the node was turned off and (+) means the node was turned on.

To verify these hypotheses, we designed two theoretical genetically modified macrophages (TGEM): HIF1-α =0 & NFκB =1 and HIF1-α =0 & STAT1 = 1. Dynamic analysis constrained by the inactivation of HIF1-α and activation of NFκB showed a significant reduction in the number of attractors, 4096, compared to the 10430 obtained initially. The HIF1-α =0 & STAT1 = 1 (STGEM) diminished the number of attractors (3840) distributed in four phenotypes (**Figure S10**). On the contrary, HIF1-α =0 & NFκB =1 had only two phenotypes (**Figure 6A**).

Interestingly, all the attractors belonged to two phenotypes, M1 and M1M2d hybrid. The M1M2d hybrid has a cytotoxic action, but the M2d counterpart diminishes the damage caused its cytotoxic function. M1 was the more stable due to its basin of attraction, while M1M2d was the least stable. We developed a cell fate map for our TGEM to acknowledge which genes can transit from one phenotype to another. Remarkably, we identified a reversible transition between M1 and M1M2d, mainly driven by Fra-1 and toll-like receptor 4 (TLR4) (**Figure 6B**). This means that our TGEM will cycle between these two phenotypes without developing new phenotypes; both are resistant to perturbations.

Our genetically modified macrophage with STAT1 activated (STGEM) developed four phenotypes. All of them had a hybrid state where the cytotoxic action was always present (**Figure S10A**). M1M2b was the most stable phenotype, while M1M2d as well as the least stable. The cell fate map of STGEM is more complicated than TGEM. The transition from M1 to M1M2b marked by STAT3 and ERK activation. STAT3 and ERK are activated by common interleukins in a tumor microenvironment like IL-10, TNF-α, and IL1β. The rest of the map is depicted in **Figure S10B**, it showed that the four phenotypes cycle between them without any new phenotype. Based on this information, STGEM may be used as a strategy for eliminating tumor cells. Due to the nature and simplicity of the phenotypes obtained in TGEM, it can be an optimal option for an immunotherapeutic strategy to modulate the tumor microenvironment to eliminate tumor cells.

Finally, we concluded that our *in silico* TGEM (conceptually defined by the permanent activation and inactivation of NFκB and HIF1-α, respectively) induced phenotypes against cancer cells in the tumor microenvironment. We predicted balanced M1 characteristics that liberate all the components involved in the elimination phase of cancer immunosurveillance and the substances to resolve inflammation and tissue damage caused by cancer. Therefore, generating an M1/M2 ratio with a good prognosis. Furthermore, the phenotypes coming from our TGEM were robust, only certain transcription factors could redirect the polarization to another phenotype (**Figure 6B**).

### Theoretical Genetically Modified Macrophage Resisted Perturbations in a Breast Cancer Microenvironment

Given the experimental evidence and previous results of the intricate and determining relationship of the microenvironments in macrophage polarization, we developed an *in-silico* microenvironment based on experimental evidence. We decided to simulate the alterations in the microenvironment of our previous TGEM. Microenvironment alterations are associated with adverse prognosis in other studies (43). We defined the state (active or inactive) of all the extracellular nodes present at the cancer microenvironment using cancer multi-omics data (44). We calculated the differential expressed genes between breast cancer cells and healthy tissue to estimate which extracellular nodes are highly present and then fixed the state of all extracellular nodes in our network. In the particular case of components such as immunoglobulin G (39), adenosines (45), glucocorticoids (46), and under hypoxia (47). All these are activated (node state:one) due to previous reports supporting their activity in samples of patients with breast cancer. Overall, we simulated our theoretical genetically modified macrophage (TGEM) in four different simplified breast cancer microenvironments to evaluate its behavior and determine if this pharmaceutical approach could be suitable. Under this microenvironment, we analyzed the effect of IgG and A2a; IL10 and TGF-β; IL-1β and IL 6; Hypoxia and glucocorticoids.

**Figure 7:**
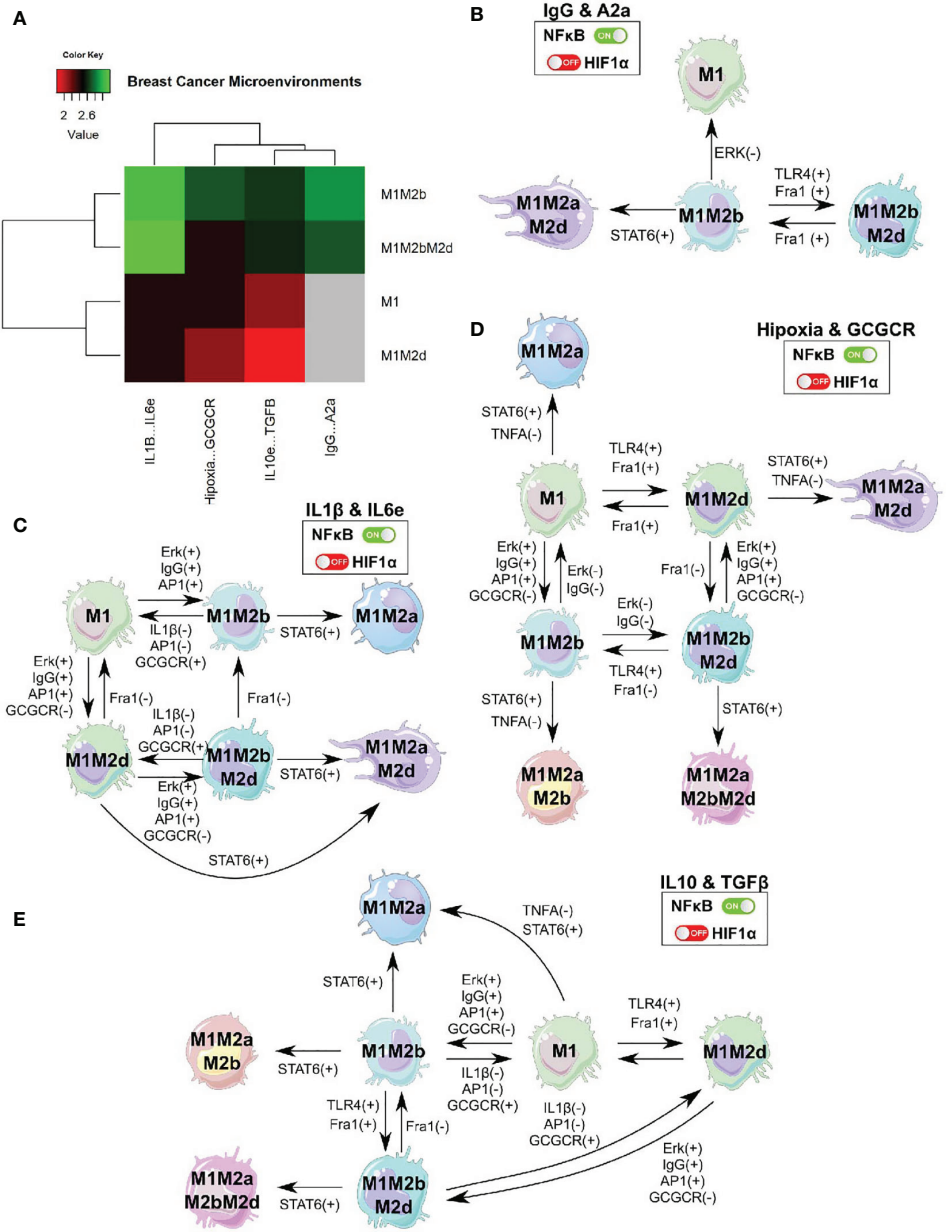
TGEM in a breast cancer microenvironment. **(A)** We engineered different microenvironments associated with breast cancer and evaluate how our TGEM behaved. **(B)** Cell fate map of the theoretical genetically modified macrophage in breast cancer microenvironment for the expression of IgG and adenosines. **(C)** Cell fate map of the theoretical genetically modified macrophage in breast cancer microenvironment for the expression of IL1-β and IL-6. **(D)** Cell fate map of the theoretical genetically modified macrophage in breast cancer microenvironment for the expression of Hypoxia and glucocorticoids (GCGCR). **(E)** Cell fate map of the theoretical genetically modified macrophage in breast cancer microenvironment for the expression of IL-10 and TGF-β. This analysis was to evaluate the stability of our pharmaceutical approach in a breast cancer scenario.

First, we evaluated the behavior modification of our TGEM in specific breast cancer microenvironments according to previous conditions. The phenotypes obtained changed globally, we found two new phenotypes: M1M2b and M1M2bM2d. Both phenotypes have the M1 function creating a cytotoxic/regulatory microenvironment (**Figure 7A**). The most complicated microenvironment (this microenvironment is implicated with metastasis) was the one with the presence of A2a and Ig G. We observed only two phenotypes: M1M2b and M1M2bM2d, while M1 and M1M2d were absent. This analysis suggested that IgG & A2a could inhibit functions of the M1 macrophage through the inactivation of TLR4 and consequently the activation of NFκB. A2a can inhibit NFκB as well; both components created a complicated microenvironment for the development of more M1 type macrophages.

Nevertheless, the phenotypes generated in this microenvironment can help eliminate tumor cells and balance the microenvironment to a more suitable one to create anti-tumoral macrophages. The remaining microenvironments develop four macrophage phenotypes (M1, M1M2b, M1M2bM2d, and M1M2d) with different proportions each. Therefore, we found a high proportion of pure M1 and M1 hybrid to shift the balance towards a tumor eliminating microenvironment, and having the regulatory components not cause more damage from the cytotoxic activity (**Figure 7A**).

In **Figures 7B–E**, we depict the cell fate map of our TGEM under the four breast cancer microenvironments evaluated. Overall, once STAT6, is activated, induce an irresistible into the phenotypes by adding the M2a components. Even though in the IgG & A2a environment, we did not have the M1 phenotypes, they can be irreversibly obtained by inhibiting the function of Erk in the M1M2b phenotypes. The M1M2b and M1M2bM2d phenotypes create feedback between them by activating Fra1 or TLR4 to transit from M1M2b to M1M2bM2d; this transition can be reversible by inhibiting the expression of Fra1. This complicated microenvironment in our TGEM may be an obstacle. However, from the phenotypes obtained and the one (M1) obtained, our TGEM may still eliminate tumor cells beside the microenvironment (**Figure 7B**).

In IL1β & IL6 microenvironment, M1 and M12b developed feedback between them where the transitions are reversible. This feedback may create a balance in the environment where the immune system can eliminate tumor cells and recuperate the damaged tissue caused by the cytotoxic functions (**Figure 7C**). Under hypoxic conditions (**Figure 7D**), our TGEM still develops macrophages with the capacity to eliminate tumor cells. The lack of oxygen does not affect the feedback between M1-M1M2b and M1M2d-M1M2bM2d. In this hypoxic microenvironment, the activation of STAT6 may serve as an adaptation agent. STAT6 is associated with the regulation of the lipid metabolism, our macrophages can obtain energy from lipids to liberate cytotoxic interleukins and eliminate tumor cells. From a theoretical perspective, STAT6 activation in our TGEM in a hypoxic scenario is not one we want to avoid (48, 49). Finally, in **Figure 7E**, a microenvironment with regulatory activity in macrophage cytotoxic functions does not affect creating phenotypes to eliminate tumor cells. M1 and M1M2b develop the same feedback as the previous microenvironment. M1M2bM2d and M1M2d create feedback between them; therefore, the transitions are reversible.

In summary, the Boolean modeling of the TRN mimicking the feasible space of macrophage phenotypes serves as a platform to create hypotheses of the control mechanism that promotes cancer phenotype. We postulate that TGEM associated with pro-inflammatory is a promising pharmaceutical approach because it is robust to permanent perturbation in a breast cancer microenvironment. This hypothesis has to be experimentally proven, an aim that constitutes a perspective of this paper.

### Robustness and Sensitivity Analysis of Our Transcriptional Regulatory Network of Macrophage Polarization in a Tumor Microenvironment

A robust gene regulatory network is resistant to perturbations in the network. We defined robustness as a characteristic of behavior invariance, according to changes in their inner variables. Nevertheless, gene regulatory networks or any biological network representing the phenotypic behavior of cells have to gain an ability to respond appropriately to external stimuli and filter molecular perturbations.

**Figure 8:**
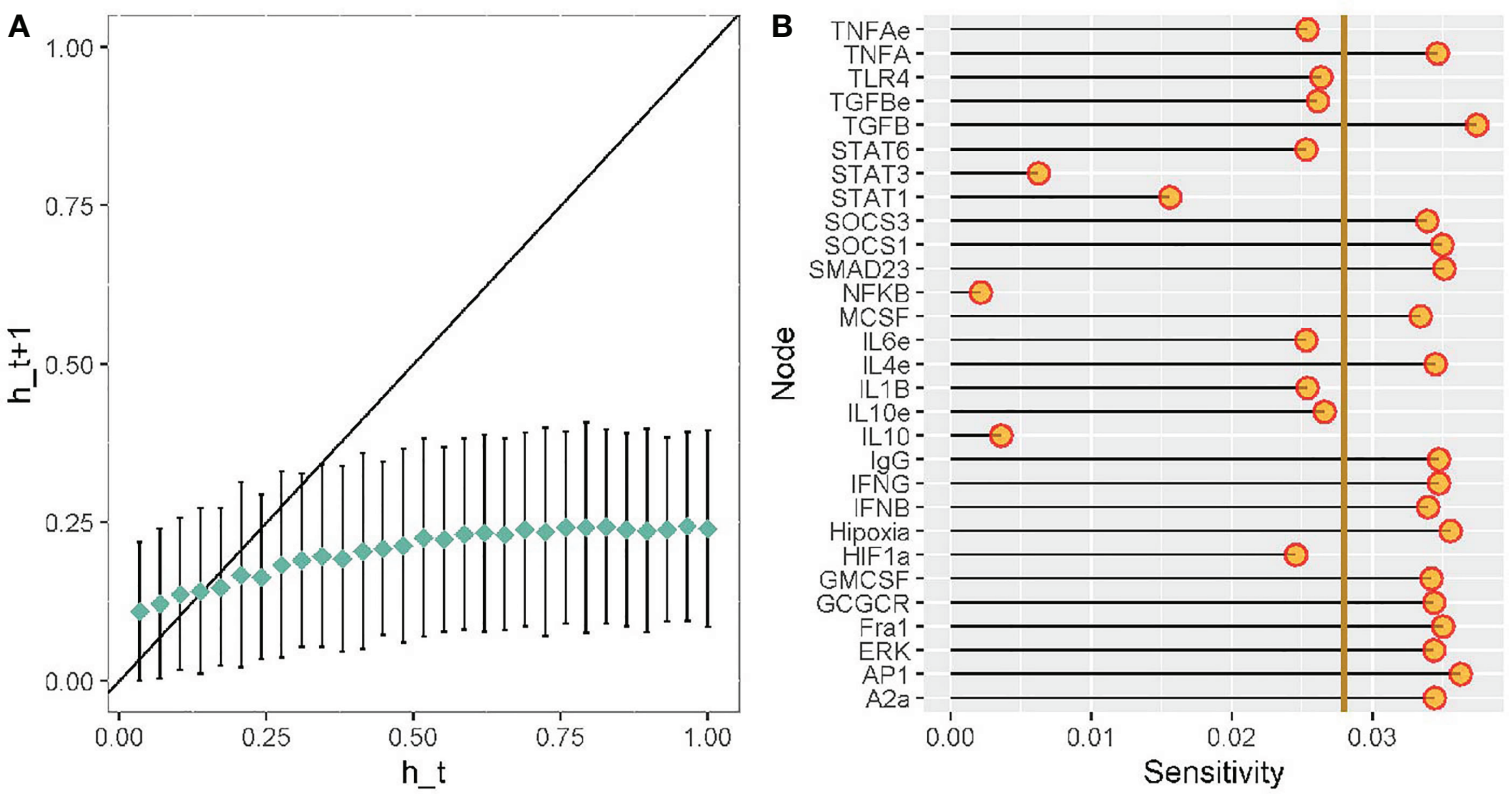
Robustness analysis of our transcriptomic regulatory network of macrophage polarization. Sensitivity analysis of each node from our transcriptional regulatory network of macrophage polarization. **(A)** Derrida curves of our transcriptional regulatory network of macrophage polarization in a tumor microenvironment. **(B)** Sensitivity analysis of the variables in our transcriptional regulatory network of macrophage polarization in a tumor microenvironment. Golden line is the mean of the value of the sensitivity analysis.

A mathematical representation of a gene regulatory network can be dynamically classified as ordered, critical, or chaotic regime. Ordered networks are not affected by filter external stimuli. Critical networks are intermediate from an ordered and chaotic behaviors, they are resilient to a specific subset of perturbations. Finally, chaotic networks are severely affected by perturbations, inducing a plethora of responses (50). To evaluate the dynamical behavior of macrophage polarization in our TRN we obtained the Derrida curve, see **Figure 8A**. To determine in what extend the slope of the curve around x=0 differ from identity line and thus classify the dynamic behavior of our network, we applied a chi-squared Goodness of fit test with the coordinates of the average Hamming values before the intersection point between both curves. We consider the observed values as those average Hamming distances depicted in the y-axis of the plot. Simultaneously, we considered the expected values as the average Hamming distances outlined in the identity line (y=x). Despite our models seeming to draw a curve slightly above the identity line around x=0, our statistical test allowed us to conclude that there is no significant difference in the Hamming distance between the observed and the expected Hamming distances (exact multinomial test of goodness-of-fit, p-value=0.5804, statistical significance= 0.05). Given that there was not a statistical difference between expected and observed values, we supplied evidence that the dynamic behavior of our network falls into the critical region. There is evidence that criticality is a trademark for living systems for simultaneously co-exist in robustness and evolvability capacities (51, 52). This finding suggests that TRN for macrophage polarization in the tumor microenvironment seems to be robust to perturbations and permits evolve according to the environmental cues.

To evaluate the sensitivity in our TRN of macrophage polarization, we modeled multiple perturbations of each update rule. The sensitivity analysis of a gene or node evaluates the influence of the other genes of our network. **Figure 8B** outlines the average sensitivity of every node and the entire network (golden line). TGF-β and IFN-γ are the nodes with higher sensitivity, followed by glucocorticoid receptor (GCGCR), interleukin 4 (IL-4), and suppressor of cytokine signaling 1 (SOCS1). Consequently, the extracellular cytokines more susceptible to stimuli or molecular noise. For example, IFN-γ is associated with an anti-tumoral microenvironment and a cytotoxic M1 macrophage function. Contrary, TGF-β is associated with pro-tumoral microenvironment and a regulatory wound healing M2 phenotypes. TGF-β is associated with inhibiting M1 functions and enhance tumor proliferation. GCGCR and IL-4 are associated with regulatory and wound healing behavior as well. SOCS1 can inhibit STAT1 and diminish the expression of IFN-γ in the microenvironment. NFκB, STAT1, and STAT3 have lower average sensitivity, thus, they are robust to transient changes. Therefore, we acknowledged NFκB and STAT1 as a therapeutic strategy for immunotherapy in tumor eradication. The network’s average sensitivity is lower than one, hence our network behaves orderly (**Figure 8A**).

## Discussion

The interaction between the immune system, microenvironment, and cancer is one appealing topic to design effective treatments. Moreover, the development of computational approaches that contribute to clarify their mechanisms is a needed task. By and extensive Boolean analysis, we presented a high quality curated signaling regulatory network between cancer-derived factors and macrophages. Unlike previous reports, we present a model with additional interactions experimentally validated and an in-depth analysis of the macrophages’ hybrid phenotypes. As Palma et al. previously concluded, these intermediary steady-states support the hypothesis that macrophage polarization is a continuum process and not binary, as usually suggested. Furthermore, our extended analysis allowed us to identify phenotypes that suggested new physiological interpretations associated with hybrid macrophage phenotypes. Based on our results, we concluded that the hybrid phenotypes evolve constrained by a permanent interaction with the environment. We postulate that some of them have therapeutic implications by enhancing tumoricidal capacity (M1M2d and M1M2b) or promoting regulatory mechanisms (M2bM2d, M2aM2d, and M2cM2d) against cancer.

Notably, our analysis allowed us to build hypotheses towards the hybrid states that favor or contrast cancer phenotype. We concluded that M1M2d, a tumoricidal-regulatory macrophage, could potentially eliminate tumor cells due to the secretion of cytotoxic cytokines and IL-12, the latest helps to differentiate CD4+ T cells. As we know, M1 is an essential player in host defense, but if it is not regulated, it could cause tissue damage. Nonetheless, the M2d part of the hybrid phenotype will secrete IL-10 and TGF-β. These regulatory cytokines would eventually maintain at bay the action caused by the cytotoxic cytokines. The M1M2d phenotype would induce a cytotoxic/regulatory cytokine ratio that could eradicate tumor cells and avoid tissue damage. Furthermore, M1M2b has a similar function as the M1M2d. It would regulate with IL-10 the damage caused by the cytotoxic actions. However, it would eventually eliminate tumor cells through the action of M1 and heal the damage caused by the elimination of tumor cells aside from the action of M2b.

Contrastingly, M2bM2d, M2aM2d, and M2cM2d are hybrid states with a malignant phenotype that express HIF1α. When a tumor grows and increases its diameter, the oxygen supply becomes insufficient in inner regions, creating hypoxic or necrotic areas (47). Hypoxia response *via* HIF1α not only affects macrophage polarization (53) also recruits macrophages and mesenchymal stromal cells to these regions (54, 55). All three macrophages phenotypes have different actions on tumor progression. For example, M2bM2d would favor the angiogenic process with the contribution of vascular endothelial growth factor (VEGF-A).

Meanwhile, the M2aM2d hybrid would be the most dangerous phenotype because it could heal the “wound” caused by tumor growth. Moreover, M2aM2d would favor tumor angiogenesis by secreting PDGF, TGF-β, IL-8, CXCL12, and VEGF-A, thus contributing to tumor angiogenesis metastasis. A shred of additional experimental evidence describes this hypoxia adaptation is given by M2a, which inhibits T cell expansion, reducing tumor clearance (56). Finally, M2cM2d and other malignant hybrids would be spatially constrained into a hypoxic area, behaving as regulators secreting IL-10 and TGF-β, creating a tumor proliferation scenario progression. Hypothetically, the inhibition of IL-10 or the coactivation of CD40, IL-12, IL-8, and TNF-α, could repolarize these macrophages to the M1 phenotype, where reverse tumor development has been demonstrated (57). Overall, we postulate that the behavior of these macrophages hybrids is malignant, given their characteristics they could promote carcinogenesis and regulate the immune system in favor of tumor phenotype (58, 59).

Recent advances have shed the different cells that constitute specialized niches and the mechanisms that promote cell-to-cell interaction. The knowledge of the cellular content and diversity of the tumor microenvironment in malignant transformation and other metastatic diseases is relevant. Most human tissues, including the breast, sustain their continuous replenishment from primary stem cells. The microenvironment that maintains homeostasis promotes cell differentiation according to functional demands and suppresses aberrant cells’ potential emergence. The interaction can be direct or bystander between malignant and non-malignant macrophages presumably powerfully influence the disease outcome, and cytokines secretion by macrophages is a fundamental factor for epithelial to mesenchymal transition (EMT) in breast cancer. Whether they promote retention of primitive cells within their niches to avoid mobilization into bloodstream or external tissues, as occurs in bone marrow, stills a matter in question (57, 60). Even though it is beyond this paper’s scope, it is becoming clearer the critical role of the M1/M2 macrophage polarization in the malignant progression of TNBC. These malignant macrophage hybrids are mostly going to express type II cytokines, promoting cancer stems cells (CSC) growth. M2 macrophages protect CSC from the immune surveillance mechanisms and induce anti-inflammatory microenvironments that plays as onco-promoters. Additionally, M2 macrophage contribute to drug resistance, particularly at the late stages of tumorigenesis (57).

In our TGEM approach, we mutated NFκB and HIF1α. NFκB is a master regulator with high activation and translocation efficiency. Based on our results, it is a promising key player to eliminate tumor cells. Having vital roles in the macrophage function. The nanoparticle approach develops a shift from an M2 macrophage type to M1 (61), very similar to our TGEM approach; nevertheless, only targeting NFκB is not sufficient. Hence, we knocked out HIF1α, because this factor is affected by hypoxia, a constitutive condition in a tumor microenvironment, that triggers the M2 polarizing effect (62). In our TGEM, we obtained three hybrid phenotypes all with M1 combined with a regulatory behavior macrophage. Contrastingly under the knockout of HIF1α, the macrophages still going to migrate to hypoxic regions but not for the same reason described earlier. This time, the spatial accumulation of macrophages in the hypoxic areas is due to their ability to scavenge apoptotic cells. Additionally, the macrophages activate the secretion of IL-12 and IFN-γ generating positive feedback with CD4+ T cells that will terminate theoretically in tumor clearance. By this matter, hypoxia can no longer influence our TGEM. It will not be immobilized in hypoxia regions, contributing to an adverse prognosis in breast cancer.

Even though this approach shows good macrophage behavior in a tumor microenvironment, we must take care of the Boolean model’s limitations. There is no grading scale in the associated factors, because Boolean models only consider 0 or 1 values. So, we cannot evaluate the amount of a transcription factor is sufficient to obtain a specific macrophage subtype. As we had proven, this reconstruction includes the necessary information to explore and supply evidence of the macrophage polarization transformation. However, we should update the components and their Boolean rules (**Figure 1**), as more experimental evidence will be appearing, and draw a more accurate description of this phenomenon. Finally, we considered the M0 phenotype as a monocyte, which is a precursor of macrophage activation. However, this type of cell has heterogeneity that could be manipulated differently in cancer cells (63). We believe that the monocyte continuum or reprogramming by cancer cells can be an essential variable in determining the macrophage continuum’s behavior. This behavior should be studied for understanding completely macrophage polarization and the complexity in between. Alternatively considering the heterogeneity of the myeloid (monocytes and neutrophils) population set an appealing future perspective.

Here, we aimed to understand the molecular and external mechanisms that orchestrate macrophage polarization and develop potential therapeutic strategies. Several strategies focus on diminishing the recruitment of macrophages to the tumor site, showing the limitation of tumor vascularization and metastasis in mouse models (64). However, these treatments only reduce tumor growth by reducing the M2 cells (65), losing the beneficial properties of M1 type macrophages. We showed that our designed macrophage diminished malignant hybrid phenotypes and adapted to the perturbations caused by the tumor microenvironment. Our theoretical approach follows a combined strategy, which we believe will be the key to design potential therapies with macrophages. Our theoretical model also suggested that the best approach to defeat cancer is not shifting M2 to M1, as most pharmaceutical methods do. This shift is easily lost because it is affected by the tumor microenvironment (66). Instead, an optimal therapeutic strategy could be highlighting the best characteristics in the pro and anti-inflammatory scenarios. W concluded that the simultaneous action of HIF1α and NFκB allows us achieving “control” over the influence of hypoxia and the cytotoxic behavior of macrophages. Finally, our computational approaches may contribute to set the foundations of the macrophage population dynamics under the phases of the cancer immunoediting (elimination, equilibrium and escape), in the hallmarks of breast cancer. Future directions and remaining challenges in investigating transitional biology from immunosurveillance to suppressor macrophages will include high throughput genomic or cytometric analyses of M1 and M2 populations in breast cancer-associated tumor microenvironment. The understanding of the dynamic process from immunosurveillance to malignant progression may unravel the principles of the dual host-protective or -harmful roles of M1 and M2 macrophages in tumors immunoediting. In recap, computational modeling has become a crucial tool for paving novel avenues in immunotherapies capable to implement optimal strategies for fighting against highly invasive breast cancer.

## Data Availability Statement

The original contributions presented in the study are included in the article/**Supplementary Material**. All code used for analysis is available at https://github.com/resendislab/M1-M2-Macrophage-Polarization.

## Author Contributions

UA-P and OR-A conceived and designed the mathematical model. UA-P performed all computational analysis and analyzed the data. MM-G and AV-J helped with the analyses of the data. RP with OR-A supervised the mathematical model and the *in-silico* analysis. All authors contributed to the article and approved the submitted version

## Funding

The authors thank the financial support from PAPIIT-UNAM (IA202720). UA-P received a doctoral fellowship from Consejo Nacional de Ciencia y Tecnología (CONACYT) (CVU: 774988).

## Conflict of Interest

The authors declare that the research was conducted in the absence of any commercial or financial relationships that could be construed as a potential conflict of interest.
