# Supplementary material for: Transcriptional and Microenvironmental Landscape of Macrophage Transition in Cancer: A Boolean Analysis
Source: Front Immunol. 2021 Jun 10;12:642842. doi: 10.3389/fimmu.2021.642842 (PMC8222808; doi:10.3389/fimmu.2021.642842)
Supplement: Supplementary file 1 [file DataSheet_1.docx]

Supplementary Material

# Supplementary Figures


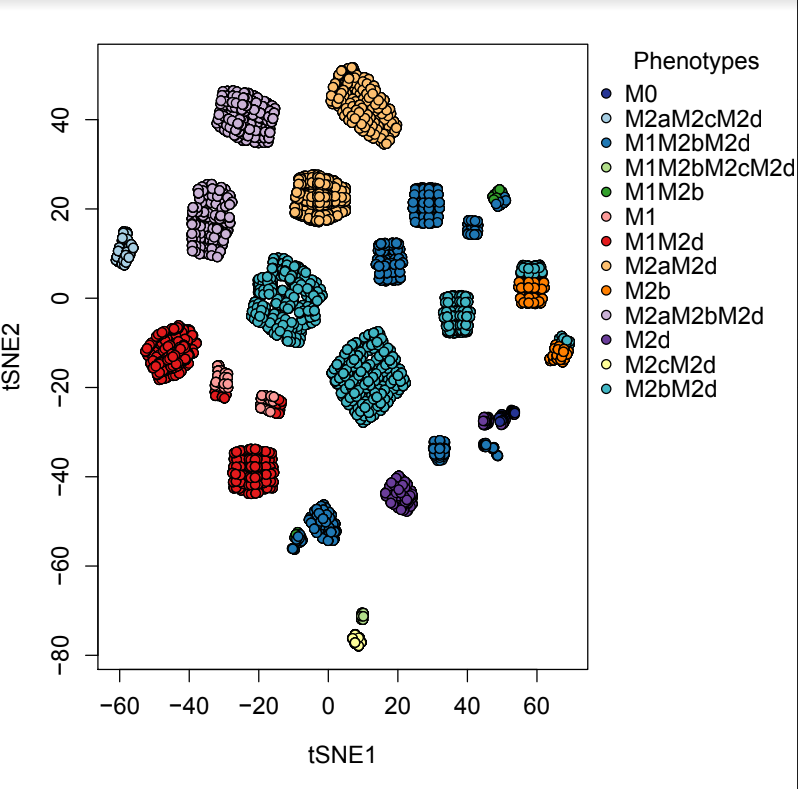


**Supplementary Figure 1. t-SNE plot of the phenotypes obtained from our network of macrophage polarization.** The attractors were handed a point in a x-y plane based on the expression of 0 and 1’s, attractors with the same combination of 0 and 1’s were placed nearby.

**
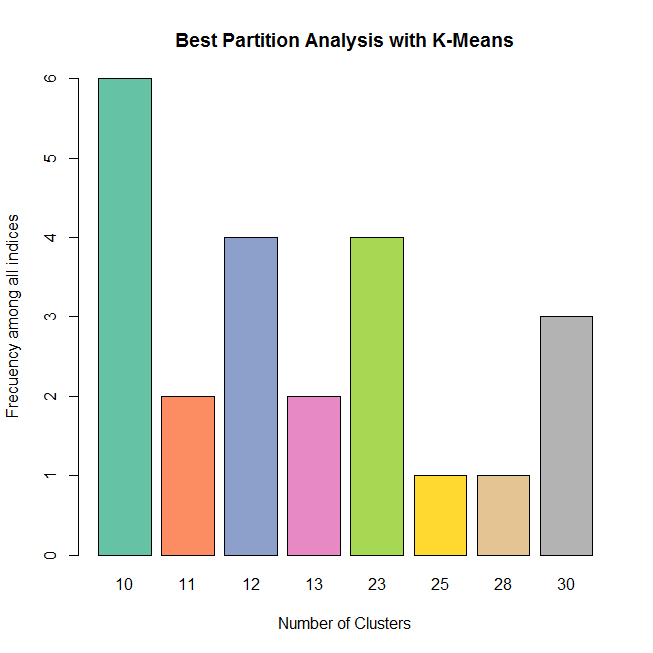
**

**Figure S2. The optimal number of clusters for K-means.** We evaluated 23 metrics to determine the number of clusters to obtain the best clusters scheme, we used the data obtained from the t-SNE points to evaluate the number of clusters that will explain the distribution of phenotypes.


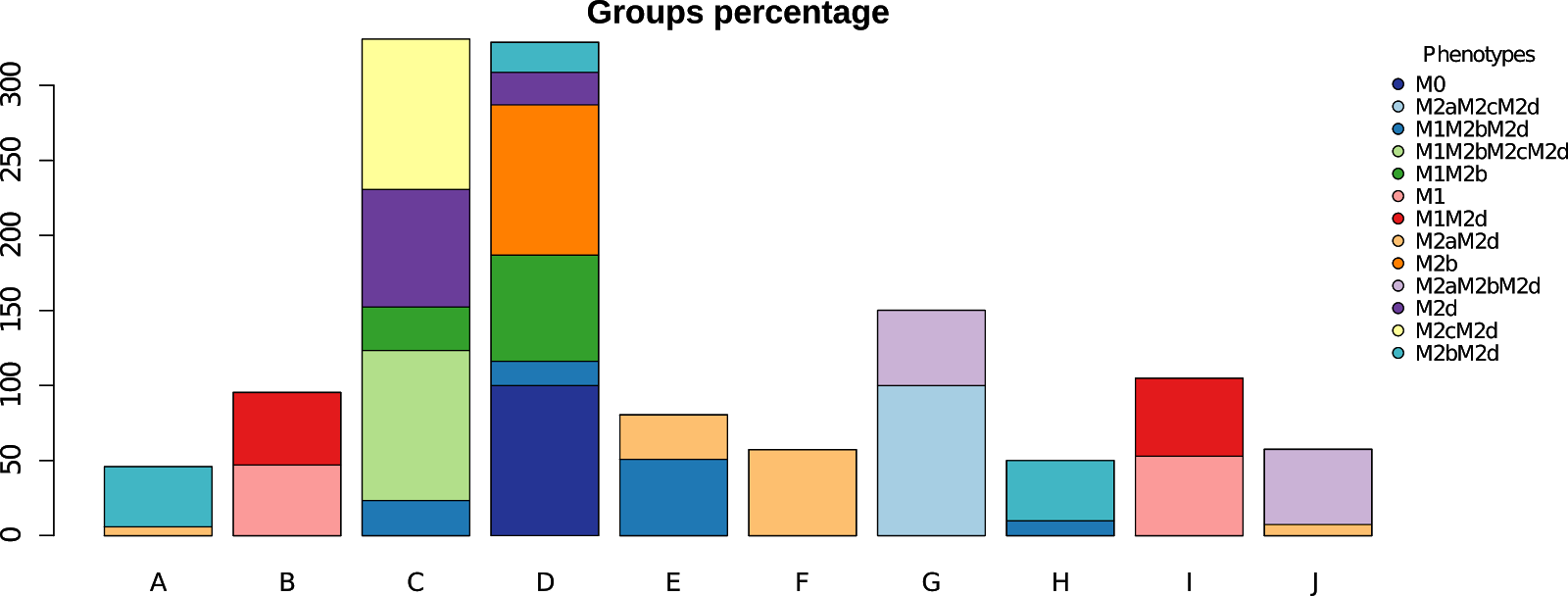


**Figure S3. Groups percentage of each phenotype in the 10 clusters used in K-means**. Each color is associated with a certain phenotype and each bar represents one cluster.

**
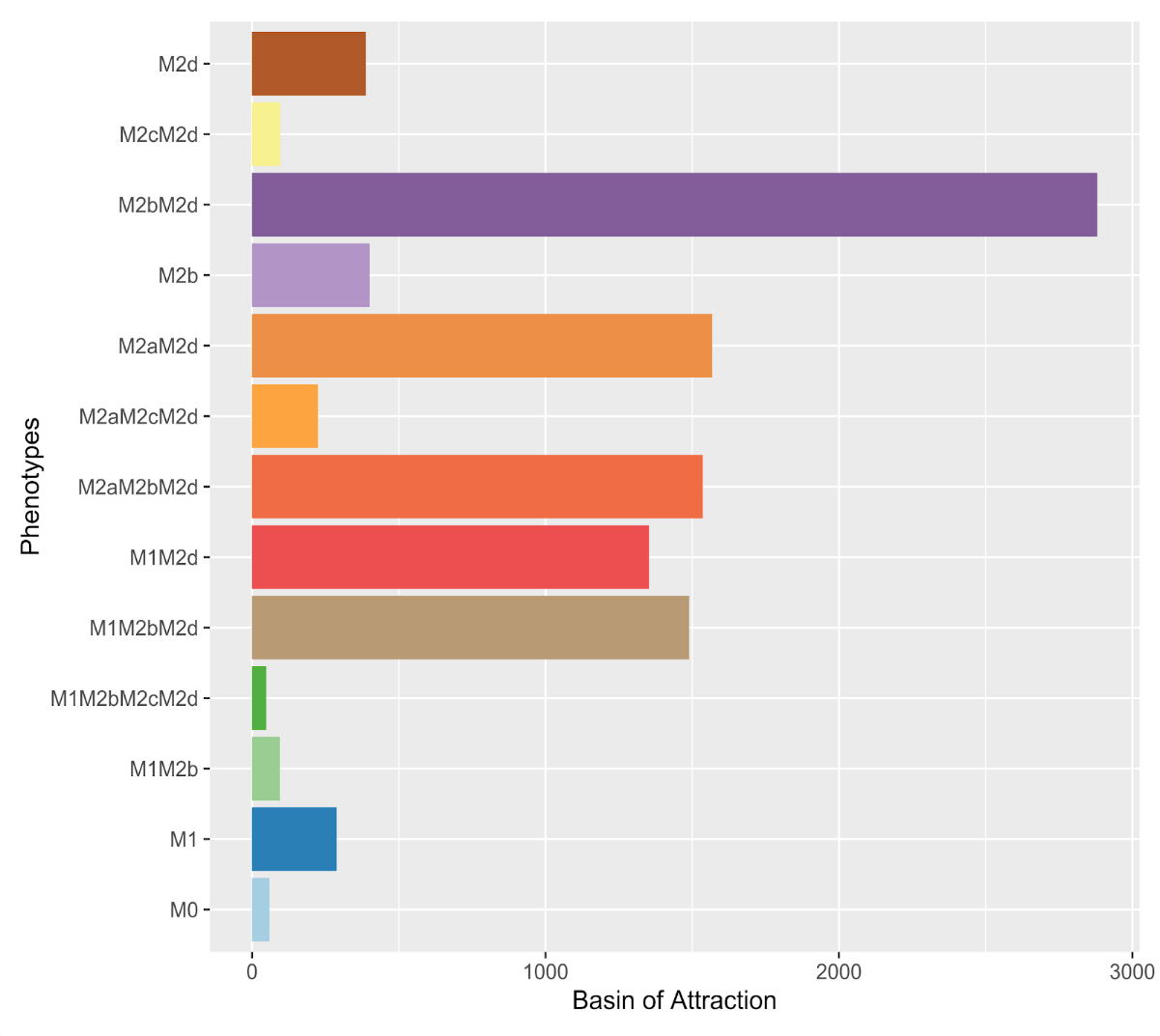
**

**Figure S4. Basin of attraction of the phenotypes obtained from our network of macrophage polarization.** Box plot of the basin of attractors obtained from our transcriptional regulatory network of macrophage polarization.We explored all the possible initial conditions based on the number of nodes 2^29^.

**
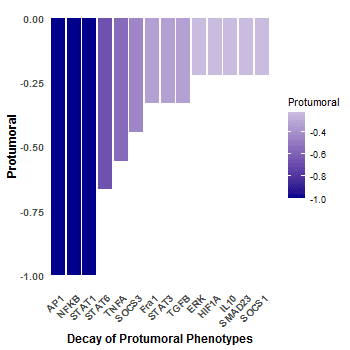
**

**Figure S5. Decay of pro-tumoral phenotypes when overexpressed transcriptional factors.** The number of pro-tumoral macrophage phenotypes depicted in figure 3A were counted. Pro-tumoral phenotypes are attractors that have a M2 macrophage phenotype but do not have a M1 phenotype. In figure 3A are 9 pro-tumoral phenotypes, we divided the number of pro-tumoral phenotypes lost (gray areas) when the perturbations were applied on said nodes.


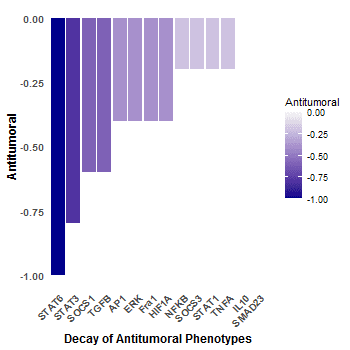


**Figure S6. Decay of anti-tumoral phenotypes when overexpressed transcriptional factors.** The number of pro-tumoral macrophage phenotypes depicted in figure 3A were counted. Pro-tumoral phenotypes are attractors that have a M2 macrophage phenotype but do not have a M1 phenotype. In figure 3A are 5 anti-tumoral phenotypes, we divided the number of anti-tumoral phenotypes lost (gray areas) when the perturbations were applied on said nodes


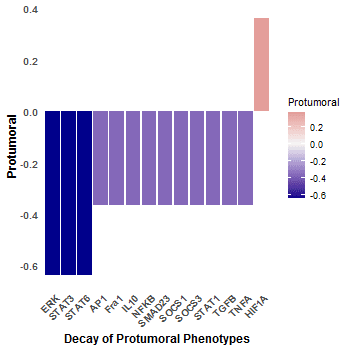


**Figure S8.**The number of pro-tumoral macrophage phenotypes depicted in figure 3B were counted. Pro-tumoral phenotypes are attractors that have a M2 macrophage phenotype but do not have a M1 phenotype. In figure 3B are 10 pro-tumoral phenotypes, we divided the number of pro-tumoral phenotypes lost (gray areas) when the perturbations were applied on said nodes.


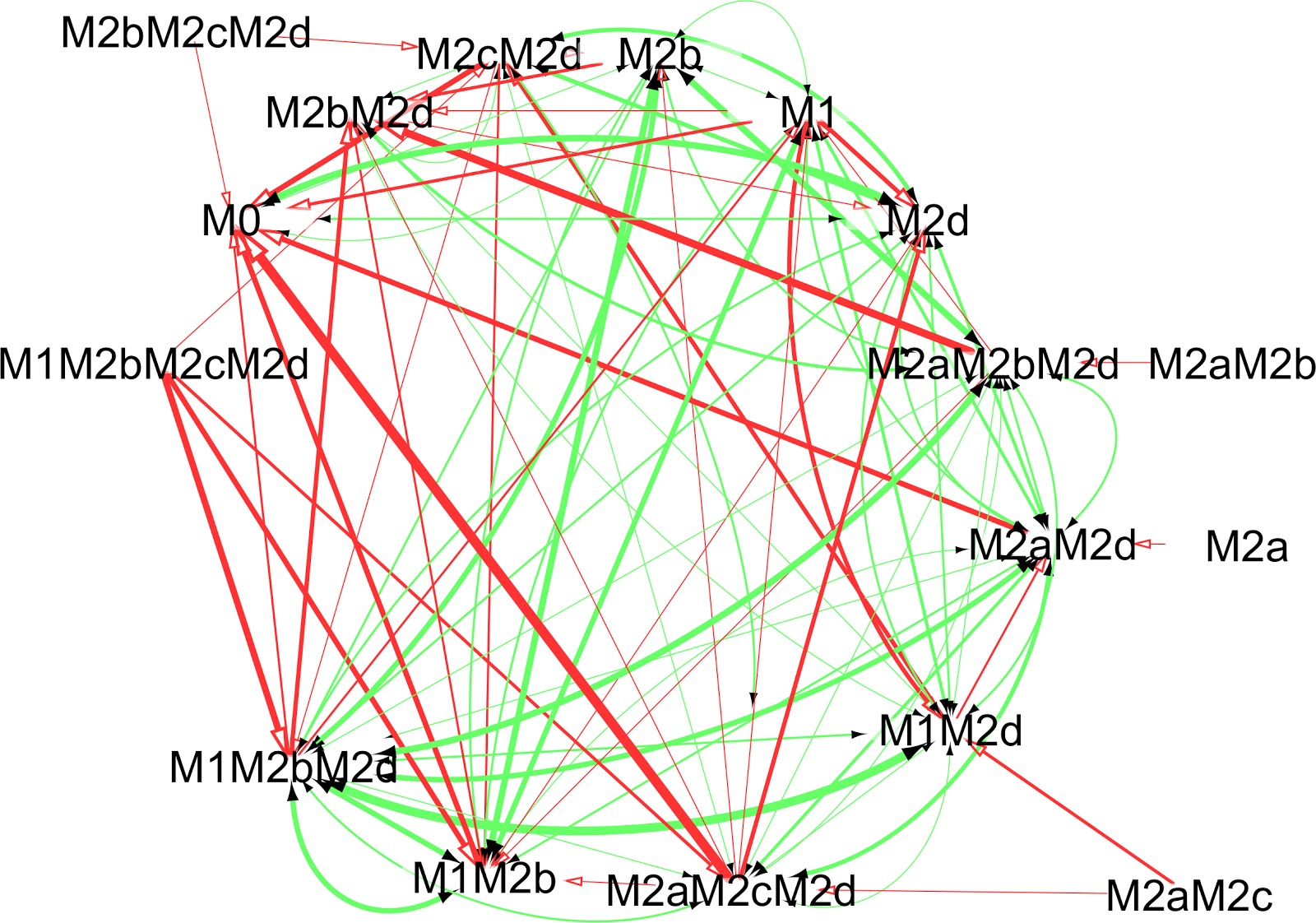


**Figure S9. Cell Fate Map of Macrophage Polarization.** Network of macrophage polarization and their interaction between phenotypes. Red color means that the transition from that phenotype to another is irreversible, meanwhile green means that is reversible. The thickness of the line means the number of nodes that are involved in said transition, the thinner the line, means that only one node is sufficient to dictate said transition between phenotypes.

**
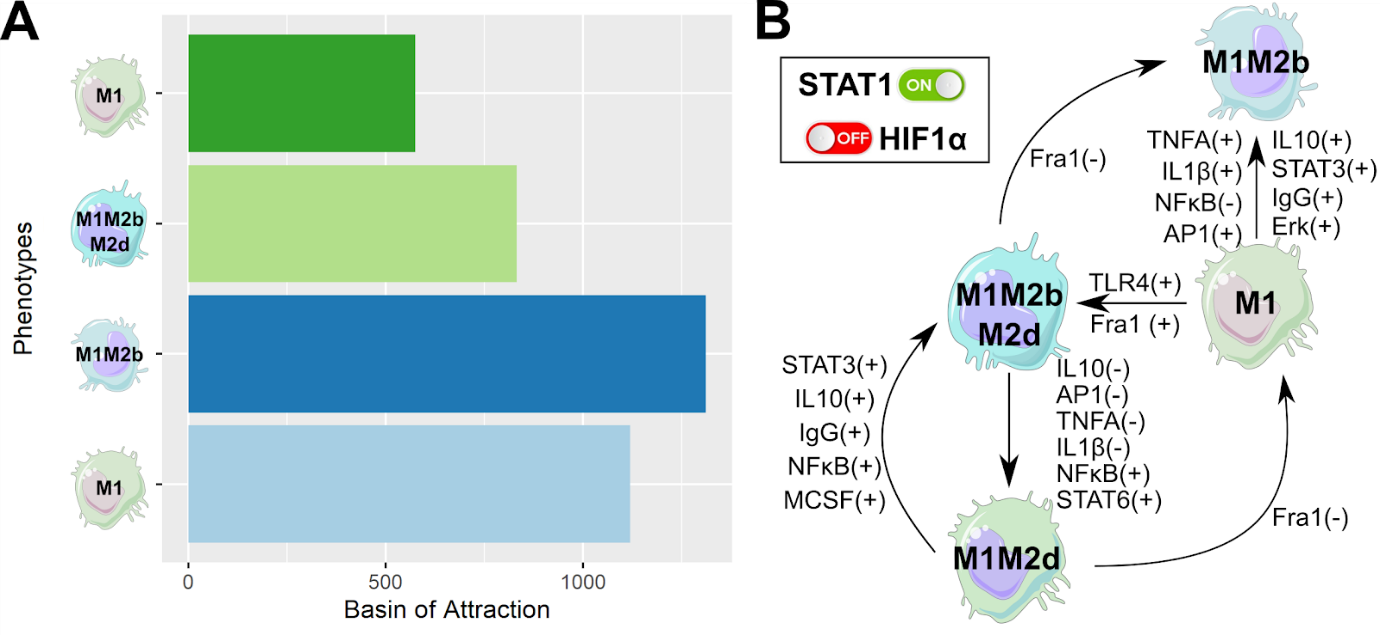
**

**Figure S10.Cell Fate Map of Macrophage Polarization.** Network of the polarization of macrophages and their interaction between phenotypes. Red color means that the transition from that phenotype to another is irreversible, meanwhile green means that is reversible. The thickness of the line means the number of nodes that are involved in said transition, the thinner the line, means that only one node is sufficient to dictate said transition between phenotypes.

# Supplementary tables

| **Node** | **Boolean Functions** | **Reference** |
| --- | --- | --- |
| STAT3(t+1) | (IL10 $\vee$ A2a $\vee$ IL10e $\vee$ (IL6e $\wedge$IL4e) $\wedge$¬ IFNG) $\vee$ ((IL6e $\wedge$ MCSF) $\vee$ MCSF) $\wedge$¬ (SOCS3 $\vee$ IFNG $\vee$ IFNB $\vee$ NFKB $\vee$GMCSF $\vee$ IgG) | [(16)](https://paperpile.com/c/cQbhD6/eWn2);[(21–24)](https://paperpile.com/c/cQbhD6/KXyc+c7OD+QkBS+DLvJ) |
| SMAD23(t+1) | (TGFBe $\vee$ TGFB) $\wedge$¬ (IFNG $\vee$ SOCS3) | [(25)](https://paperpile.com/c/cQbhD6/m5cA)[(26)](https://paperpile.com/c/cQbhD6/gFrC) |
| STAT6(t+1) | (IL4e $\vee$ SMAD23$\vee$HIF1A) $\wedge$¬ (STAT1 $\vee$ TNFA $\vee$ TNFAe) | [(26–28)](https://paperpile.com/c/cQbhD6/gFrC+iLbm+GzzU) |
| SOCS1(t+1) | STAT6 $\vee$ IgG | [(29)](https://paperpile.com/c/cQbhD6/zywQ) [(30)](https://paperpile.com/c/cQbhD6/0OOY) |
| TLR4(t+1) | TLR4 $\wedge$¬ (IgG$\vee$GCGCGR) | [(1,2)](https://paperpile.com/c/cQbhD6/SKS1+d1Yl) |
| NFKB(t+1) | (TLR4 $\vee$ GMCSF $\vee$ TNFA $\vee$ TNFAe $\vee$IL1B) $\wedge$¬ (IL10 $\vee$ IL10e $\vee$ STAT6 $\vee$ STAT3 $\vee$ IgG $\vee$ GCGCR $\vee$ HIF1A $\vee$ MCSF) | [(7,31)](https://paperpile.com/c/cQbhD6/VT6k+yaL6);[(8)](https://paperpile.com/c/cQbhD6/WARx); [(31)](https://paperpile.com/c/cQbhD6/yaL6) |
| STAT(t+1) | (IFNB $\vee$ IFNG $\vee$ (IL6e $\wedge$ IFNG)) $\wedge$¬ (STAT3 $\vee$ SOCS1 $\vee$ STAT6 $\vee$ IgG $\vee$AP1) | [(7,31)](https://paperpile.com/c/cQbhD6/VT6k+yaL6) |
| SOCS3(t+1) | STAT1 $\vee$ TLR4 | [(3,4,32)](https://paperpile.com/c/cQbhD6/dRGG+zDv1+rDfF) |
| AP1(t+1) | (GMCSF $\vee$ TNFAe $\vee$ TNFA $\vee$ IL6e $\vee$ IL1B) $\wedge$¬ (GCGCR $\vee$ STAT6) | [(33–35)](https://paperpile.com/c/cQbhD6/fBXc+nyhO+ZNAf) |
| IL10(t+1) | (IL10e $\vee$ (IL4e $\wedge$ STAT6) $\vee$ (IL6e $\wedge$ STAT3) $\vee$ (TLR4 $\wedge$ A2a) $\vee$ ERK $\vee$ MCSF) $\wedge$¬ (STAT1 $\vee$ NFKB $\vee$ SOCS3 $\vee$ IFNG $\vee$ IFNB $\vee$ GMCSF $\vee$ IgG) | [(14,36)](https://paperpile.com/c/cQbhD6/UeWT+i5HU);[(21–24)](https://paperpile.com/c/cQbhD6/KXyc+c7OD+QkBS+DLvJ);[(14)](https://paperpile.com/c/cQbhD6/UeWT)[(16)](https://paperpile.com/c/cQbhD6/eWn2)[(14)](https://paperpile.com/c/cQbhD6/UeWT) |
| TNFA(t+1) | (TNFAe $\vee$ AP1 $\vee$ (IL6e$\wedge$AP1) $\vee$ (Fra1$\wedge$AP1)) $\wedge$¬ (STAT1 $\vee$ NFKB $\vee$ (TLR4 $\wedge$A2a)) | [(33–35)](https://paperpile.com/c/cQbhD6/fBXc+nyhO+ZNAf); [(18–20)](https://paperpile.com/c/cQbhD6/9tIx+bl5v+OsVd) |
| IL4e(t+1) | IL4e | [(26–28)](https://paperpile.com/c/cQbhD6/gFrC+iLbm+GzzU) |
| TGFB(t+1) | (TGFBe $\vee$ (IL4e $\wedge$STAT6) $\vee$ (IL10e$\wedge$STAT3) $\vee$(IL10e $\wedge$ STAT3) $\vee$ A2a) $\wedge$¬ (NFKB $\vee$STAT1) | [(37,38)](https://paperpile.com/c/cQbhD6/JDYo+tORf) |
| IL10e(t+1) | IL10e $\vee$ STAT3 $\vee$ STAT6 |  |
| TGFBe(t+1) | TGFBe $\vee$ STAT3 $\vee$ STAT6 |  |
| TNFAe(t+1) | TNFAe $\vee$ AP1 |  |
| IgG(t+1) | IgG |  |
| A2a(t+1) | A2a |  |
| IFNG(t+1) | IFNG |  |
| IFNB(t+1) | IFNB $\vee$ NFKB |  |
| IL1B(t+1) | IL1B $\vee$ AP1 |  |
| Fra1(t+1) | Fra1 $\vee$ TLR4 | [(39)](https://paperpile.com/c/cQbhD6/YdQ0) |
| IL6e(t+1) | IL6e $\vee$ Fra1 $\vee$ AP1 |  |
| MCSF(t+1) | MCSF |  |
| GMCSF(t+1) | GMCSF |  |
| Hipoxia(t+1) | Hipoxia |  |
| HIF1A(t+1) | Hipoxia $\vee$ TGFB $\vee$ TGFBe $\vee$ (TLR4 $\wedge$ A2a) | [(40–42)](https://paperpile.com/c/cQbhD6/LcW1+aFvN+r8YB) |
| ERK(t+1) | IgG $\vee$ IL6e |  |
| GCGCR(t+1) | GCGCR |  |

**Table S1. Interactions and boolean functions in the macrophage polarization netwo**rk. $\wedge$: AND, $\vee$: OR, and ¬: NOT

## Molecular signaling pathways of Macrophage Polarization

Inflammation is the tissue response to an injury, an etiological event, necrosis, or other causes. This process involves interactions of cells, cytokines, chemokines, and other products that initiate a signaling cascade and cellular changes. In the early stages of cancer inflammation, mononuclear phagocytes group on the site of tumor growth. They start by releasing pro-inflammatory substances, creating an anti-tumor microenvironment, and positive feedback because these substances, once they bind to their receptors, will trigger the transcriptional machinery for the release of other pro-inflammatory cytokines developing a tumor-free microenvironment. This feedback maintains the M1 macrophage. Afterwards, tumors develop a response from the pro-inflammatory substances, liberating cytokines that down-regulate the pro-inflammatory effects of cytokines, creating a pro-tumoral microenvironment. This microenvironment switches macrophages or any other leucocyte to regulatory functions (the same behavior in the resolution phase of inflammation). This substance inhibits the M1 transcriptional program and activates the M2 program. This negative feedback ensures an anti-inflammatory program.

The toll-like receptor 4 (TLR4) is a receptor activated by lipopolysaccharide found in bacteria. Therefore, this mechanism should lack in breast tumor progression. However, the release of HMGB1 protein by dying tumor cells activates the TLR4-MyD88 signaling pathway to generate a response against the tumor cells [(1,2)](https://paperpile.com/c/cQbhD6/SKS1+d1Yl). Once activated, TLR4 induces cytokines and the expression of chemokines to initiate an anti-tumor response. Also, it generates SOCS3, which inhibits STAT3, favoring the activation of the M1 macrophage. It activates NF𝝹B, which will produce pro-inflammatory cytokines [(3,4)](https://paperpile.com/c/cQbhD6/dRGG+zDv1).

Interferon binds to its specific receptor in the cytoplasmic membrane of the macrophage. This interaction promotes a conformational change in the Janus-Kinase signal transducer (JAK). Subsequently, STAT1 is phosphorylated and dimerized. Once STAT2 is activated, it induces pro-inflammatory genes like IL-12, IL1𝛃, and iNOS. This signaling pathway is of great importance because it is present in other immune cells. It produces essential substances that tend to eliminate tumor cells for their cytotoxic activity [(5,6)](https://paperpile.com/c/cQbhD6/o7Vl+Wc02).

The macrophage colony-stimulating factor (GM-CSF) stimulates the polarization of the macrophage to the M1 phenotype. Its mechanism of action is through Jak2 that activates STAT5, NF-κB, and IRF5. Therefore, GM-CSF improves the presentation of antigen, leukocyte chemotaxis, adhesion and phagocytosis through the system of complements and antibodies, and the release of cytokines (IL-6, IL-8, G-CSF, M-CSF, TNF, and IL-1β). Due to the simplicity of our model, we only considered the activation of NF-κB. STAT5 would only produce a linearity, using more nodes without producing much information or feedback [(7,8)](https://paperpile.com/c/cQbhD6/VT6k+WARx).

The M2 phenotype is more complex than M1 because it has subtypes activated through diverse routes.

IL-4 or IL-13 activates M2a. IL-4 can bind IL-4Ralpha1 and IL-13Ralpha1, allowing the binding of IL-4 or IL-13. Once the IL-4 receptor is activated, it induces the expression of STAT6. STAT6 can directly inhibit STAT1 through the expression of SOCS1, and induces the expression of factor 4 similar to Kruppel (KLF4), which inhibits NF-κB by avoiding the binding of p50 and p65 heterodimers.

M2b is a subtype of the M2 phenotype[(9)](https://paperpile.com/c/cQbhD6/vfhV)different from M1 and M2a . This subtype is activated by the IL-1R receptor, TLR4, IL-1β, or by a damage signaling factor. Besides, M2b can be activated by immunological complexes with Fc receptors on their membrane. These cells have an opposite behavior than the M1 macrophage because they produce low levels of IL-12 and high levels of IL-10. They also have a different behavior than M2a because they produce high levels of TNF-alpha, IL-1β, and IL-6 and have the capacity to generate an adaptive type II immune response. As immunological complexes can activate it, this cell can secrete antibodies, specifically the IgG1 isotype, with a change to type II IgG. This cell expresses the enzyme sphingosine kinase; therefore, it can respond to sphingosine-1 phosphate, a factor that generates angiogenic growth.

M2c is activated by glucocorticoids and IL-10, each having a different mechanism of activation. Glucocorticoid activates its glucocorticoid alpha receptor (GCR), which binds to the transcription factors NF-κB and AP-1, affecting the inflammatory response. Glucocorticoids inhibit NF-κB inducing the expression of inhibitory protein IκBalfa, which traps NF-κB in an inactive cytoplasmic complex, avoiding the translocation to the nucleus and activating its white genes (active genes vital in response to a pro-inflammatory stimulus) [(10,11)](https://paperpile.com/c/cQbhD6/Rbxb+atza).

Transforming growth factor-beta (TGF-β) belongs to a superfamily of growth factors, presenting three isoforms. It is a type of protein involved in cellular processes from hematopoiesis to cell differentiation, having important mechanisms in cell migration and apoptosis. Several types of cells secrete TGF-β, such as endothelial cells, lymphocytes, macrophages, and tumor cells. The action mechanism of TGF-β begins when it is bound to its receptor, generating a conformational change in phosphorylated transcription factors SMAD2 and SMAD3. The SMAD2-3-4 complex is then translocated to the nucleus to over-code genes to produce anti-inflammatory cytokines such as arg1 and mgl2, generating an M2 phenotype. This pathway can be inhibited by SMAD7, which prevents the phosphorylation of SMAD2/3 or leads to its degradation; TNF-alpha or IFN-γ can activate this transcription factor; both factors are a crucial part of the M1 phenotype.

M2d is a subtype of macrophage present in solid tumors whose stimulation can be through interleukin 6 (IL-6), leukemia inhibitory factor (LIF), and adenosine. LIF induces differentiation towards M2d through the autocrine loop of IL-6 / M-CSF. In a few words, LIF favors the de novo synthesis of IL-6. LIF and IL-6 generate a higher consumption of M-CSF, favoring the reprogramming of M2 when activating IRF4 [(12)](https://paperpile.com/c/cQbhD6/lpph).

IL-6 has another mechanism to generate a pro-inflammatory response but is not as efficient as the previous one since SOCS3 can inhibit the activation of STAT3 by IL-6; therefore, this mechanism of action does not favor the induction of the phenotype M2 [(13)](https://paperpile.com/c/cQbhD6/QjTQ).

Adenosines are purine nucleosides, essential for the intracellular metabolic pathways. Cells release adenosines into the extracellular space in response to metabolic disturbances and other types of insults, such as inflammation, physical damage, and apoptosis [(14)](https://paperpile.com/c/cQbhD6/UeWT). Adenosines affect the macrophage reprogrammation towards the M2 phenotype. For example, it favors the M2a phenotype when activating STAT6 through the A2B receptor [(15)](https://paperpile.com/c/cQbhD6/aTgk).

The same adenosine also presents a regulation mechanism for reprogramming towards M2c. It increases the phosphorylation of STAT3 mediated by IL-10, therefore increases the expression of genes of this macrophage. It inhibits the activation of STAT3 by IL-6, inhibiting a pro-inflammatory response [(16,17)](https://paperpile.com/c/cQbhD6/eWn2+MZEo).

Adenosine has a crucial function in the macrophage's polarization towards an M2d phenotype known as the angiogenic change. The new characteristic of this phenotype is that it has to behave like cytotoxic M1 first, and then, it differentiates on a M2d phenotype. TLR4 has to be activated to increase the expression of a hypoxia-inducible transcriptional factor (HIF1-α) through an independent pathway of NF-κB,. HIF1-α is essential to release of VEGF (vascular endothelial growth factor). Interestingly, when TLR4 is activated, it activates the expression of the adenosine A2a receptor, which stabilizes the transcripts of HIF-1 and its proteins [(18–20)](https://paperpile.com/c/cQbhD6/9tIx+bl5v+OsVd).

References:

1. [Apetoh L, Ghiringhelli F, Tesniere A, Obeid M, Ortiz C, Criollo A, Mignot G, Maiuri MC, Ullrich E, Saulnier P, et al. Toll-like receptor 4-dependent contribution of the immune system to anticancer chemotherapy and radiotherapy. Nat Med (2007) **13**:1050–1059.](http://paperpile.com/b/cQbhD6/SKS1)

2. [Toll-like receptor stimulation in cancer: A pro- and anti-tumor double-edged sword. Immunobiology (2017) **222**:89–100.](http://paperpile.com/b/cQbhD6/d1Yl)

3. [Kawasaki T, Kawai T. Toll-Like Receptor Signaling Pathways. Frontiers in Immunology (2014) **5**: doi:](http://paperpile.com/b/cQbhD6/dRGG)[10.3389/fimmu.2014.00461](http://dx.doi.org/10.3389/fimmu.2014.00461)

4. [Liu H, Shi B, Huang C-C, Eksarko P, Pope RM. Transcriptional diversity during monocyte to macrophage differentiation. Immunol Lett (2008) **117**:70–80.](http://paperpile.com/b/cQbhD6/zDv1)

5. [Sica A, Mantovani A. Macrophage plasticity and polarization: in vivo veritas. J Clin Invest (2012) **122**:787–795.](http://paperpile.com/b/cQbhD6/o7Vl)

6. [Long KB, Collier AI, Beatty GL. Macrophages: Key orchestrators of a tumor microenvironment defined by therapeutic resistance. Mol Immunol (2019) **110**:3–12.](http://paperpile.com/b/cQbhD6/Wc02)

7. [Martinez FO, Gordon S. The M1 and M2 paradigm of macrophage activation: time for reassessment. F1000Prime Rep (2014) **6**:13.](http://paperpile.com/b/cQbhD6/VT6k)

8. [Krausgruber T, Blazek K, Smallie T, Alzabin S, Lockstone H, Sahgal N, Hussell T, Feldmann M, Udalova IA. IRF5 promotes inflammatory macrophage polarization and TH1-TH17 responses. Nat Immunol (2011) **12**:231–238.](http://paperpile.com/b/cQbhD6/WARx)

9. [Charles F. Anderson & David M. Mosser. A novel phenotype for an activated macrophage: the type 2 activated macrophage. Journal of Leuckocyte Biology (2002) **72**:101–106.](http://paperpile.com/b/cQbhD6/vfhV)

10. [Auphan N, DiDonato JA, Rosette C, Helmberg A, Karin M. Immunosuppression by glucocorticoids: inhibition of NF-kappa B activity through induction of I kappa B synthesis. Science (1995) **270**:286–290.](http://paperpile.com/b/cQbhD6/Rbxb)

11. [Herrlich P. Cross-talk between glucocorticoid receptor and AP-1. Oncogene (2001) **20**:2465–2475.](http://paperpile.com/b/cQbhD6/atza)

12. [Labonte AC, Tosello-Trampont A-C, Hahn YS. The Role of Macrophage Polarization in Infectious and Inflammatory Diseases. Molecules and Cells (2014) **37**:275–285.](http://paperpile.com/b/cQbhD6/lpph)

13. [Yasukawa H, Ohishi M, Mori H, Murakami M, Chinen T, Aki D, Hanada T, Takeda K, Akira S, Hoshijima M, et al. IL-6 induces an anti-inflammatory response in the absence of SOCS3 in macrophages. Nat Immunol (2003) **4**:551–556.](http://paperpile.com/b/cQbhD6/QjTQ)

14. [Haskó G, Pacher P. Regulation of Macrophage Function by Adenosine. Arteriosclerosis, Thrombosis, and Vascular Biology (2012) **32**:865–869.](http://paperpile.com/b/cQbhD6/UeWT)

15. [Csóka B, Selmeczy Z, Koscsó B, Németh ZH, Pacher P, Murray PJ, Kepka-Lenhart D, Morris SM Jr, Gause WC, Leibovich SJ, et al. Adenosine promotes alternative macrophage activation via A2A and A2B receptors. FASEB J (2012) **26**:376–386.](http://paperpile.com/b/cQbhD6/aTgk)

16. [Koscsó B, Csóka B, Kókai E, Németh ZH, Pacher P, Virág L, Leibovich SJ, Haskó G. Adenosine augments IL-10-induced STAT3 signaling in M2c macrophages. J Leukoc Biol (2013) **94**:1309–1315.](http://paperpile.com/b/cQbhD6/eWn2)

17. [Scheller J, Chalaris A, Schmidt-Arras D, Rose-John S. The pro- and anti-inflammatory properties of the cytokine interleukin-6. Biochim Biophys Acta (2011) **1813**:878–888.](http://paperpile.com/b/cQbhD6/MZEo)

18. [Ramanathan M, Luo W, Csóka B, Haskó G, Lukashev D, Sitkovsky MV, Leibovich SJ. Differential regulation of HIF-1alpha isoforms in murine macrophages by TLR4 and adenosine A(2A) receptor agonists. J Leukoc Biol (2009) **86**:681–689.](http://paperpile.com/b/cQbhD6/9tIx)

19. [Pinhal-Enfield G, Ramanathan M, Hasko G, Vogel SN, Salzman AL, Boons G-J, Leibovich SJ. An angiogenic switch in macrophages involving synergy between Toll-like receptors 2, 4, 7, and 9 and adenosine A(2A) receptors. Am J Pathol (2003) **163**:711–721.](http://paperpile.com/b/cQbhD6/bl5v)

20. [Ferrante CJ, Pinhal-Enfield G, Elson G, Cronstein BN, Hasko G, Outram S, Leibovich SJ. The adenosine-dependent angiogenic switch of macrophages to an M2-like phenotype is independent of interleukin-4 receptor alpha (IL-4Rα) signaling. Inflammation (2013) **36**:921–931.](http://paperpile.com/b/cQbhD6/OsVd)

21. [Hutchins AP, Diez D, Miranda-Saavedra D. The IL-10/STAT3-mediated anti-inflammatory response: recent developments and future challenges. Brief Funct Genomics (2013) **12**:489–498.](http://paperpile.com/b/cQbhD6/KXyc)

22. [Martinez FO, Sica A, Mantovani A, Locati M. Macrophage activation and polarization. Front Biosci (2008) **13**:453–461.](http://paperpile.com/b/cQbhD6/c7OD)

23. [Gordon S, Martinez FO. Alternative Activation of Macrophages: Mechanism and Functions. Immunity (2010) **32**:593–604.](http://paperpile.com/b/cQbhD6/QkBS)

24. [O’Farrell AM, Liu Y, Moore KW, Mui AL. IL-10 inhibits macrophage activation and proliferation by distinct signaling mechanisms: evidence for Stat3-dependent and -independent pathways. EMBO J (1998) **17**:1006–1018.](http://paperpile.com/b/cQbhD6/DLvJ)

25. [Massagué J. TGFβ in Cancer. Cell (2008) **134**:215–230.](http://paperpile.com/b/cQbhD6/m5cA)

26. [Malyshev I, Malyshev Y. Current Concept and Update of the Macrophage Plasticity Concept: Intracellular Mechanisms of Reprogramming and M3 Macrophage “Switch” Phenotype. Biomed Res Int (2015) **2015**:341308.](http://paperpile.com/b/cQbhD6/gFrC)

27. [Kratochvill F, Neale G, Haverkamp JM, Van de Velde L-A, Smith AM, Kawauchi D, McEvoy J, Roussel MF, Dyer MA, Qualls JE, et al. TNF Counterbalances the Emergence of M2 Tumor Macrophages. Cell Rep (2015) **12**:1902–1914.](http://paperpile.com/b/cQbhD6/iLbm)

28. [Jiang Q, Akashi S, Miyake K, Petty HR. Lipopolysaccharide induces physical proximity between CD14 and toll-like receptor 4 (TLR4) prior to nuclear translocation of NF-kappa B. J Immunol (2000) **165**:3541–3544.](http://paperpile.com/b/cQbhD6/GzzU)

29. [Whyte CS, Bishop ET, Rückerl D, Gaspar-Pereira S, Barker RN, Allen JE, Rees AJ, Wilson HM. Suppressor of cytokine signaling (SOCS)1 is a key determinant of differential macrophage activation and function. J Leukoc Biol (2011) **90**:845–854.](http://paperpile.com/b/cQbhD6/zywQ)

30. [Zhou D, Chen L, Yang K, Jiang H, Xu W, Luan J. SOCS molecules: the growing players in macrophage polarization and function. Oncotarget (2017) **8**:60710–60722.](http://paperpile.com/b/cQbhD6/0OOY)

31. [Tugal D, Liao X, Jain MK. Transcriptional Control of Macrophage Polarization. Arteriosclerosis, Thrombosis, and Vascular Biology (2013) **33**:1135–1144.](http://paperpile.com/b/cQbhD6/yaL6)

32. [Hu X, Ivashkiv LB. Cross-regulation of Signaling Pathways by Interferon-γ: Implications for Immune Responses and Autoimmune Diseases. Immunity (2009) **31**:539–550.](http://paperpile.com/b/cQbhD6/rDfF)

33. [Liu Y-C, Zou X-B, Chai Y-F, Yao Y-M. Macrophage polarization in inflammatory diseases. Int J Biol Sci (2014) **10**:520–529.](http://paperpile.com/b/cQbhD6/fBXc)

34. [Liu Y, Cao X. The origin and function of tumor-associated macrophages. Cellular & Molecular Immunology (2015) **12**:1–4.](http://paperpile.com/b/cQbhD6/nyhO)

35. [Foey AD. “Immune Response Activation,” in Immune Response Activation (IntechOpen).](http://paperpile.com/b/cQbhD6/ZNAf)

36. [Lawrence T, Natoli G. Transcriptional regulation of macrophage polarization: enabling diversity with identity. Nat Rev Immunol (2011) **11**:750–761.](http://paperpile.com/b/cQbhD6/i5HU)

37. [Rhee I. Diverse macrophages polarization in tumor microenvironment. Arch Pharm Res (2016) **39**:1588–1596.](http://paperpile.com/b/cQbhD6/JDYo)

38. [Rőszer T. Understanding the Mysterious M2 Macrophage through Activation Markers and Effector Mechanisms. Mediators Inflamm (2015) **2015**:816460.](http://paperpile.com/b/cQbhD6/tORf)

39. [Wang Q, Ni H, Lan L, Wei X, Xiang R, Wang Y. Fra-1 protooncogene regulates IL-6 expression in macrophages and promotes the generation of M2d macrophages. Cell Res (2010) **20**:701–712.](http://paperpile.com/b/cQbhD6/YdQ0)

40. [Leblond MM, Gérault AN, Corroyer-Dulmont A, MacKenzie ET, Petit E, Bernaudin M, Valable S. Hypoxia induces macrophage polarization and re-education toward an M2 phenotype in U87 and U251 glioblastoma models. Oncoimmunology (2016) **5**:e1056442.](http://paperpile.com/b/cQbhD6/LcW1)

41. [Ke X, Chen C, Song Y, Cai Q, Li J, Tang Y, Han X, Qu W, Chen A, Wang H, et al. Hypoxia modifies the polarization of macrophages and their inflammatory microenvironment, and inhibits malignant behavior in cancer cells. Oncol Lett (2019) **18**:5871–5878.](http://paperpile.com/b/cQbhD6/aFvN)

42. [Raggi F, Pelassa S, Pierobon D, Penco F, Gattorno M, Novelli F, Eva A, Varesio L, Giovarelli M, Bosco MC. Regulation of Human Macrophage M1-M2 Polarization Balance by Hypoxia and the Triggering Receptor Expressed on Myeloid Cells-1. Front Immunol (2017) 8:1097.](http://paperpile.com/b/cQbhD6/r8YB)
